# Hibernation-Promoting Factor Sequesters Staphylococcus aureus Ribosomes to Antagonize RNase R-Mediated Nucleolytic Degradation

**DOI:** 10.1128/mBio.00334-21

**Published:** 2021-07-13

**Authors:** Anna Lipońska, Mee-Ngan F. Yap

**Affiliations:** a Department of Microbiology-Immunology, Northwestern University Feinberg School of Medicine, Chicago, Illinois, USA; KUMC

**Keywords:** ribosome, hibernation, RNase, stress response, *Staphylococcus aureus*

## Abstract

Bacterial and eukaryotic hibernation factors prevent translation by physically blocking the decoding center of ribosomes, a phenomenon called ribosome hibernation that often occurs in response to nutrient deprivation. The human pathogen Staphylococcus aureus lacking the sole hibernation factor HPF undergoes massive ribosome degradation via an unknown pathway. Using genetic and biochemical approaches, we find that inactivating the 3′-to-5′ exonuclease RNase R suppresses ribosome degradation in the Δ*hpf* mutant. *In vitro* cell-free degradation assays confirm that 30S and 70S ribosomes isolated from the Δ*hpf* mutant are extremely susceptible to RNase R, in stark contrast to nucleolytic resistance of the HPF-bound 70S and 100S complexes isolated from the wild type. In the absence of HPF, specific S. aureus 16S rRNA helices are sensitive to nucleolytic cleavage. These RNase hot spots are distinct from that found in the Escherichia coli ribosomes. S. aureus RNase R is associated with ribosomes, but unlike the E. coli counterpart, it is not regulated by general stressors and acetylation. The results not only highlight key differences between the evolutionarily conserved RNase R homologs but also provide direct evidence that HPF preserves ribosome integrity beyond its role in translational avoidance, thereby poising the hibernating ribosomes for rapid resumption of translation.

## INTRODUCTION

Ribosome hibernation is a widespread survival strategy among bacteria and eukaryotes. Although hibernation factors are structurally distinct, they all function to trap ribosomes in a translationally incompetent state by occupying the decoding sites of the 70S (in bacteria) and 80S (in eukaryotes) complexes (1–9). The majority of bacteria, including *Firmicutes* Staphylococcus aureus and Bacillus subtilis, harbor a long form of hibernation-promoting factor (HPF) that dimerizes the 70S monomers to form hibernating 100S ribosomes (10–12). In stark contrast to *Firmicutes*, Escherichia coli and some gammaproteobacteria require both ribosome modulation factor (RMF) and a short form of HPF (formerly YhbH) to stimulate the formation of 100S complexes. These species often harbor a third hibernation factor, YfiA (also known as pY or RaiA, a homolog of MPY in mycobacteria [13, 14]), that silences the 70S ribosome without 70S dimerization (15). Recently, HPF homologs of unclear function were found in bacteriophages (16, 17). The mammalian equivalent of 100S, a dimer of 80S monomers (110 complex), has only been observed in tumor cells under nutrient starvation (18). Adding to the dissimilarity, eukaryotic hibernation factors (Lso2/CCDC124 and Stm1/SERBP1) clamp the 40S and 60S subunits together to form an inactive 80S complex (6, 7, 19).

S. aureus HPF is one of the predominant proteins induced upon host cell internalization and during infections (20, 21), and the virulence of the *hpf* knockout (Δ*hpf*) is attenuated by 3 orders of magnitude in a murine model of infection (22). S. aureus HPF consists of a self-dimerizing C-terminal domain (CTD) and a translational silencing N-terminal domain (NTD). The protein binds to only the 30S portion of the 70S monomer and not to the 50S subunit. While the NTD-HPF blocks the mRNA channel and tRNA binding to the A-site and P-site of the decoding center, the CTD-HPF on one 70S directly interacts with another CTD-HPF that is tethered to the opposite copy of the 70S, resulting in "side-to-side" conjoining of the two 30S subunits (11, 12). There is no contact between the RMF and the short form HPF in E. coli; instead, RMF allosterically induces a "back-to-back" joining of 70S monomers at the 30S-30S interface to form the 90S dimer, followed by the short HPF-mediated stabilization of the 100S ribosome and translational inhibition resembling that of a CTD-HPF (15, 23). To reactivate hibernating ribosomes for translation and exit from dormancy, S. aureus 100S ribosomes are disassembled into recyclable 30S and 50S subunits or 70S complexes by either the RRF/EF-G pathway or through the heat-induced GTPase HflX (24, 25).

Despite striking differences in the conformation of 100S complexes and the mechanism of 70S dimerization, the loss of RMF or HPF homologs leads to convergent phenotypes, including decreases in long-term viability and regrowth (26–28), and reductions in antibiotic and stress tolerance (10, 29–31). Bacterial ribosome degradation is often triggered by nutrient downshift (32–34). However, a fraction of ribosomes is also degraded during exponential growth (33–37). More than 70% of rRNA are degraded in the Δ*hpf* strains of S. aureus (27), E. coli (38), Pseudomonas aeruginosa (39, 40) and Mycobacterium tuberculosis (13, 41), but not in B. subtilis, although its ribosome content was modestly reduced (26, 42). Rather, the essential 30S ribosomal proteins S2 and S3 are depleted from the ribosomes in B. subtilis Δ*hpf* (42), providing the first clue that hibernation protects ribosomes from damage. In either case, the RNase(s) and/or protease(s) responsible for the ribosome breakdown in the Δ*hpf* background was not identified.

Unlike other HPF homologs that are specifically expressed during the stationary phase, the S. aureus *hpf* (and 100S ribosomes) is produced as early as the lag phase, and its concentrations continue to rise, peak, and plateau following a typical bacterial growth profile, as previously confirmed by time course immunoblotting and mass spectrometry analyses of HPF-bound ribosomes (27, 43–45). Beyond this basic phenomenology, the physiological role of 70S dimerization remains obscure, although hibernating ribosomes are thought to function as storage sites to preserve translationally competent ribosomes from engaging in unscheduled translation or from degradation. The former may be less significant, because deleting HPF only moderately derepresses translation of a small subset of mRNAs (27, 46, 47). To gain insight into the latter possibility, we genetically knocked out 13 annotated S. aureus RNase-encoding genes in a Δ*hpf* strain with the rationale that if HPF-ribosome interactions interfere with a specific ribosome degradation pathway, an RNase-Δ*hpf* double mutant will restore ribosome levels to the wild-type (WT) condition or slow degradation. Here, we show that inactivating *rnr* (encodes exonuclease RNase R) significantly stabilizes ribosomal pools in a Δ*hpf* strain. S. aureus RNase R is ribosome bound and posttranslationally modified, and it preferentially cleaves the 30S subunit over the 50S subunit. Cell-free degradation assays support a model by which HPF binding protects the ribosomes from RNase R action. In the absence of HPF, several helices in the 16S rRNA are cleaved. These results collectively offer a causative link between HPF function and ribosome turnover.

## RESULTS

### Inactivation of RNase R suppresses rapid ribosome degradation in an *hpf* knockout.

**FIG 1:**
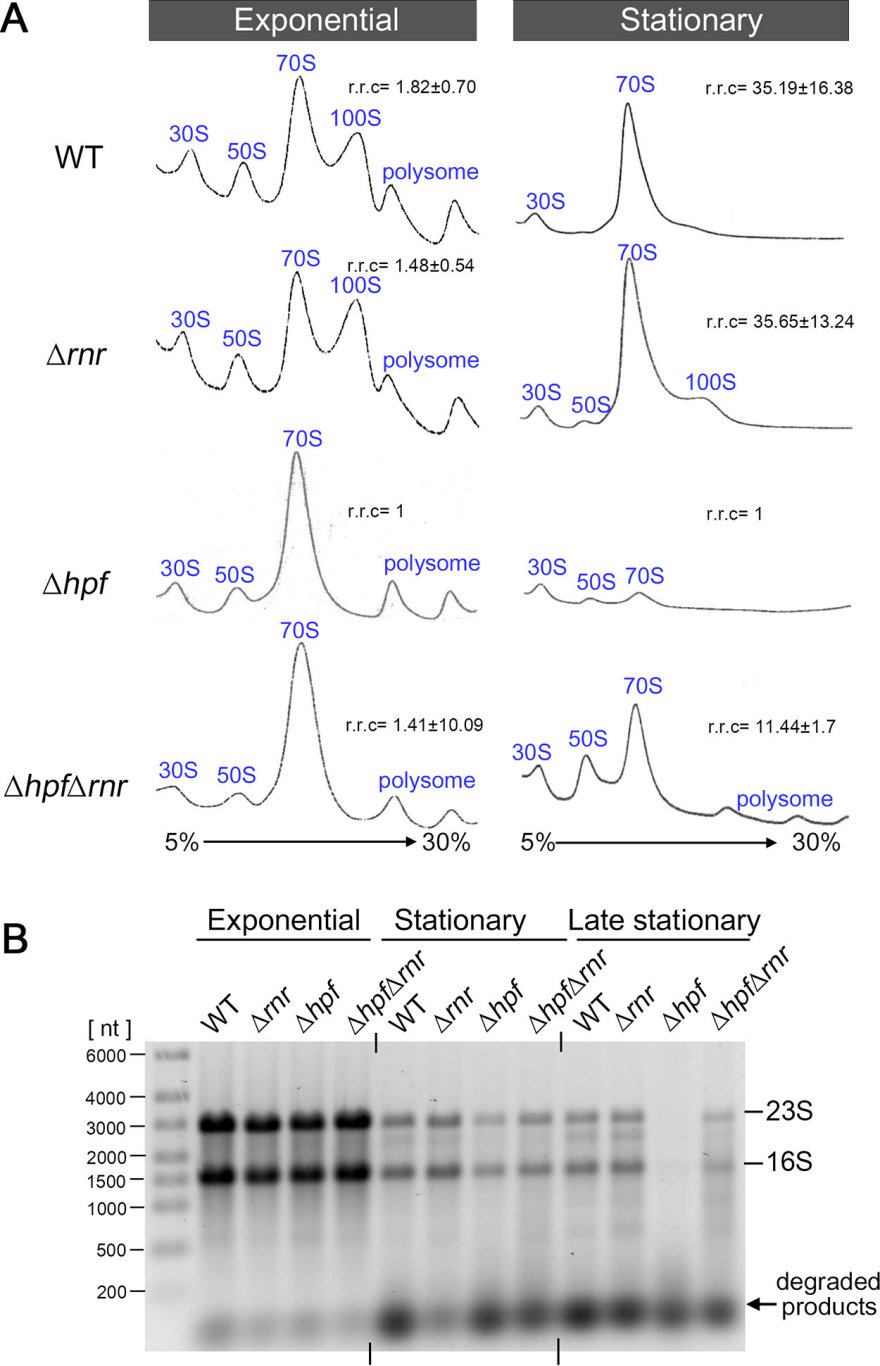
Inactivation of *rnr* suppresses ribosome degradation in the S. aureus Δ*hpf* mutant. (A) Ribosome sedimentation profiles of Δ*hpf* and Δ*rnr* single and double mutants showing the recovery of ribosome populations in a Δ*hpf* Δ*rnr* double mutant relative to the Δ*hpf* mutant (right). Crude ribosomes were isolated from TSB cultures grown until late exponential phase (OD_600_ of 1.7 to 1.8 at 37°C) or stationary phase (18 to 20 h growth at 37°C). The samples were centrifuged in a 5% to 30% sucrose gradient (*x* axis), and ribosome profiles were monitored via the absorbance at 254 nm (*y* axis). Each panel represents 5 *A*_260_ units of RNA input. Relative ribosomal content (r.r.c) was obtained from two (exponential phase) and three (stationary phase) independent biological replicates using the Δ*hpf* mutant as a reference (set as 1) according to the mean ± standard deviation (SD). To obtain r.r.c values, the areas under the peaks were quantitated by ImageJ, and total ribosomal areas were divided by that of an Δ*hpf* mutant. (B) Analysis of total RNA integrity from different growth stages. Both 23S rRNA and 16S rRNA are more prone to degradation during stationary and post-stationary phases in the Δ*hpf* knockout than in the WT or a Δ*hpf* Δ*rnr* double mutant. Three micrograms of total RNA was analyzed on a 0.8% TAE denaturing agarose gel and stained with ethidium bromide. The image shows a representative experiment of two independent biological replicates.

We used a clinically relevant methicillin-resistant S. aureus (MRSA) USA300 isolate as a model for the study of translational regulation. Bacterial ribosomes are composed of ∼60% rRNA and ∼40% ribosomal proteins. Motivated by the observation that low viability of the Δ*hpf* strain is associated with rapid ribosome turnover, we hypothesized that HPF shields ribosomes from an unidentified RNase and/or that HPF-bound ribosomes adopt a conformation that is inaccessible to the action of an RNase. To identify such an RNase, we introduced null mutations of 13 RNase genes, one at a time, to the Δ*hpf* background, reasoning that inactivation of the RNase candidate will significantly slow ribosome degradation. Ribosome sedimentation profiles were analyzed by sucrose gradient density ultracentrifugation to separate 30S, 50S, 70S, and 100S complexes, and the amount of each species was quantitated based on the areas under the curve and using the same total ribosome input (Fig. 1A). The RNases were chosen according to one or more of the following criteria: they are known to cleave structured RNA, they are involved in processing tRNA or rRNA, they are ribosome associated, mRNAs are not their major targets, and they are nonessential and readily amenable to genetic knockout (48, 49).

Among the 13 genes (*rnhA*, *mrnC*, *rnr*, *rnhC*, *rnmV*, *ybeY*, *pnpA*, *rnc*, *yefM1*, *yoeB1*, *yefM2*, *rae1*, and *mazF*) (see Table S1 in the supplemental material), only a combination of Δ*rnr* and Δ*hpf* significantly increased ribosome pools compared to a diminishment of ribosomes in the Δ*hpf* single mutant for cells collected from the stationary phase (Fig. 1A, right; see also Fig. S1). Ribosomes harvested from exponentially grown cultures were analyzed in parallel to ensure that defects in ribosome assembly did not lead to misinterpretation (Fig. 1A, left; Fig. S1). For instance, YbeY is required for 16S rRNA maturation and ribosome quality control (50). The S. aureus Δ*hpf* Δ*ybeY* mutant exhibited severe growth defects, accumulated 50S subunits, and failed to mitigate ribosome degradation; thus, the Δ*ybeY* mutant was not investigated further (Fig. S1). Bacterial RNase R is a 3′-to-5′ processing exoribonuclease that cleaves linear and double-stranded RNA (dsRNA) with 7- to 10-nucleotide (nt) 3′ overhangs without sequence specificity (49, 51, 52). Notably, PNPase is a 3′-to-5′ exoribonuclease that is functionally redundant with RNase R, but deletion of *pnpA* did not rescue the ribosome pools (Fig. S1), suggesting that catalysis directions of the enzyme are not critical and that ribosome decay is primarily driven by RNase R. However, introducing a Δ*rnr* allele did not fully restore the ribosome levels to the WT (Fig. 1A, right), implying the involvement of an unidentified secondary RNase in ribosome turnover. Furthermore, deletion of *Streptomyces rnc*, which encodes the dsRNA endonuclease RNase III, causes an accumulation of 100S ribosomes (53). S. aureus Δ*rnc* did not increase the abundance of 100S ribosomes or reduce ribosome degradation (Fig. S1), suggesting that ribosomes are not the direct substrates of RNase III.

10.1128/mBio.00334-21.1 TABLE S1 Strains and plasmids. Download Table S1, DOCX file, 0.1 MB. Copyright © 2021 Lipońska and Yap. 2021 Lipońska and Yap. https://creativecommons.org/licenses/by/4.0/ This content is distributed under the terms of the Creative Commons Attribution 4.0 International license.

10.1128/mBio.00334-21.4 FIG S1 Ribosome sedimentation profiles of ribonuclease mutants that do not restore ribosome loss in the Δ*hpf* knockout. Crude ribosomes were extracted from TSB cultures from the late exponential phase (37°C, OD_600_ of 1.6 to 1.8) or stationary phase (18 to 20 h at 37°C). Ribosomal complexes were analyzed by 5% to 30% sucrose density gradient ultracentrifugation (*x* axes). *y* axes show absorbance at 254 nm. Each panel represents 5 *A*_260_ units of RNA input. The Δ*hpf* Δ*ybeY* double mutant is severely impaired in ribosome assembly (30S-50S subunit joining), as indicated by an unusually high 50S peak and low 70S abundance. Download FIG S1, TIF file, 2 MB. Copyright © 2021 Lipońska and Yap. 2021 Lipońska and Yap. https://creativecommons.org/licenses/by/4.0/ This content is distributed under the terms of the Creative Commons Attribution 4.0 International license.

Consistent with the ribosome profiles, the integrity of rRNAs isolated from the Δ*hpf* Δ*rnr* double mutant was comparable to that of the WT isolated from the stationary phase, whereas approximately 50% of rRNAs from the Δ*hpf* single mutant was degraded (Fig. 1B). The difference was even more evident during late stationary phase, during which all rRNAs were degraded in the Δ*hpf* mutant, while >50% of rRNAs remained stable in the Δ*hpf* Δ*rnr* double mutant (Fig. 1B). Based on the genetic analyses, we conclude that HPF-free ribosomes are the targets of RNase R.

### Inactivation of *rnr* in an *hpf* knockout impairs cell growth.

Bacterial RNase R is involved in both the maturation and degradation of tRNAs and rRNAs. To determine how unintended ribosome degradation and/or accumulation of unprocessed RNA precursors affects cell growth, we compared the bacterial growth (see Fig. S2) and the doubling times of WT, Δ*hpf*, Δ*rnr*, and Δ*hpf* Δ*rnr* strains (Table 1). We found that the Δ*rnr* single knockout posed no major growth impairment, whereas the Δ*hpf* Δ*rnr* double mutant was more severely impaired than the WT and Δ*hpf* strains. These results indicate that insufficient processing and degradation by RNase R in the absence of HPF are toxic.

**TABLE 1:** Doubling time of S. aureus Δ*hpf* and Δ*rnr* mutants in TSB cultures grown at 37°C

S. aureus strain genotype	Doubling time (min)^*a*^
WT	32.0 ± 1.7
Δ*hpf*	32.4 ± 1.6
Δ*rnr*	33.1 ± 0.8
Δ*hpf* Δ*rnr*	36.9 ± 1.7

a Values are the averages from three independent experiments (means ± SDs).

10.1128/mBio.00334-21.5 FIG S2 Growth curves of the wild-type, *rnr*, and *hpf* mutants in tryptic soy broth at 37°C. A delay in cell growth was observed in the Δ*hpf* Δ*rnr* double mutant, consistent with the measurements of doubling time (see Table 1). Error bars indicate the standard errors from three replicates. Download FIG S2, TIF file, 0.1 MB. Copyright © 2021 Lipońska and Yap. 2021 Lipońska and Yap. https://creativecommons.org/licenses/by/4.0/ This content is distributed under the terms of the Creative Commons Attribution 4.0 International license.

### The expression of S. aureus RNase R is not significantly altered by growth phase and cold shock, and the protein is methylated.

**FIG 2:**
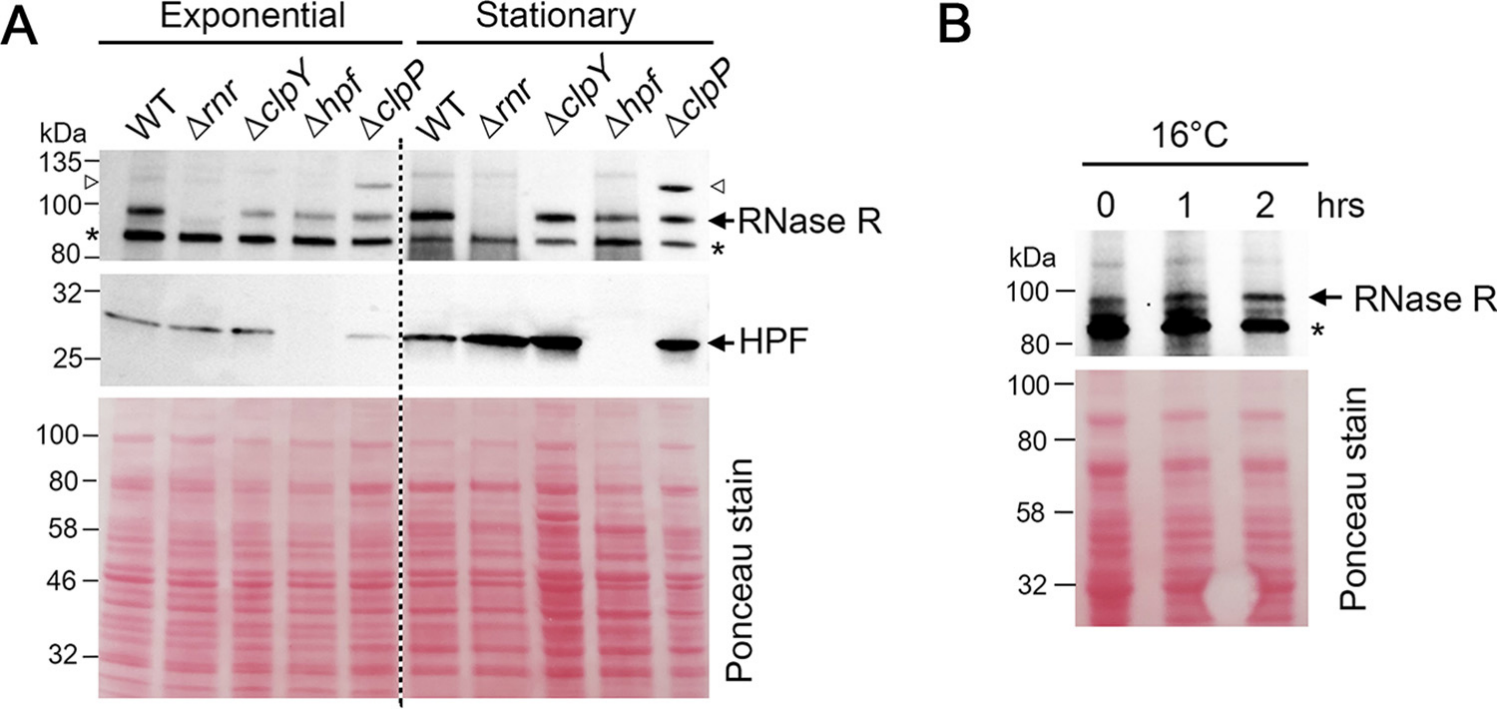
The expression profiles of S. aureus RNase R under stress conditions. (A) Western blot showing the expression of RNase R during the exponential (OD_600_ of ∼0.8 at 37°C) and stationary phases (18 to 20 h growth at 37°C). RNase R levels were not significantly altered throughout the growth phase. Neither a Δ*clpY* nor a Δ*clpP* mutant increased RNase R stability. Immunoblotting against anti-HPF served as a reference. An open triangle indicates either a nonspecific cross-reactivity or a potentially posttranslationally modified RNase R that migrates similarly to a weak band present in the WT. An asterisk marks a nonspecific band. (B) Cold shock (16°C) does not promote the expression of *rnr.* For panels A and B, each lane corresponds to 0.1 *A*_280_ units of total lysate. Proteins were resolved on a 4% to 20% TGX SDS-PAGE gel and anti-Rnr and anti-HPF antibodies were used at 1:1,000 and 1:5,000 dilutions, respectively. Ponceau S staining of the membranes prior to immunoblotting served as the loading control.

The cellular concentration of E. coli RNase R increases dramatically in response to starvation during stationary phase or cold shock that stabilizes *rnr* transcripts (54). E. coli RNase R is acetylated at residue K544, and acetylated RNase R is routed for destruction by the ClpYQ (HslUV) and Lon proteases during exponential growth. During the stationary phase, the lysine acetylase Pat (formerly Pka or YfiQ) is not expressed, leading to an increase level of RNase R (55). Three distinct features were observed between E. coli and S. aureus RNase R. First, S. aureus RNase R was detectable as early as during logarithmic growth, and the levels remained constant when cells entered the stationary phase (Fig. 2A). Importantly, deletion of *hpf* moderately downregulated RNase R, confirming that rapid ribosome degradation in the Δ*hpf* mutant is not caused by an increased concentration of RNase R. Cold shock (16°C) also did not promote *rnr* expression (Fig. 2B). Second, S. aureus RNase R was not stabilized in a *clpY* or a *clpP* null strain, suggesting that S. aureus RNase R is not a substrate of the major ClpYQ and ClpXP proteases. Finally, mass spectrometry analyses of immunoprecipitated FLAG-tagged RNase R from S. aureus revealed that the protein carries different modifications. Notably, the equivalent K544 position of E. coli RNase R is not universally conserved and is replaced by a Q537 in S. aureus RNase R (see Fig. S3A and B). S. aureus RNase R(Q537) cannot be acetylated, but a glutamine can structurally mimic (although not always) an acetyl lysine (56, 57). We found that the adjacent Q538 is methylated, as shown by an increase in the 14-Da methyl group (Fig. S3B). How the acetylation mimic influences RNase R stability and how methylation at Q538 affects RNase activity remain to be explored.

10.1128/mBio.00334-21.6 FIG S3 Comparison of RNase R homologs and their posttranslational modification sites. (A) Partial sequence alignment of representative RNase R homologs. Sequences were extracted from NCBI GenBank or UniProtKB with the following accession numbers: Escherichia coli, UniProtKB P21499; Streptococcus pneumoniae, UniProtKB C6GKN7; Bacillus subtilis, UniProtKB O32231; Campylobacter jejuni, GenBank WP_002869357.1; Mycoplasma genitalium, UniProtKB P47350; and Staphylococcus aureus, GenBank ABD22003.1. An asterisk indicates a nonconserved site that is acetylated in E. coli RNase R (residue K544). A solid circle marks the methylated Q538 of the S. aureus homolog. (B) Annotated MS/MS spectra and fragment ion assignments of Q538-methylated peptide (SMQQ^Me^AHYDDVNLGHFGLSA). Positive mass shift of 14 Da is highlighted with triple filled circles. FLAG-tagged RNase R was overexpressed on a plasmid in the S. aureus Δ*rnr* strain and immunoprecipitated. The protein band was excised from an SDS-PAGE gel and subjected to LC-MS/MS analyses. Tandem mass spectra were searched against S. aureus USA300 UniProtKB database (2,608 entries) using Mascot (Matrix Science, London, UK; version 2.7.0.1). Mascot was searched with a fragment ion mass tolerance of 0.050 Da and a parent ion tolerance of 10.0 ppm. Carbamidomethyl of cysteine was specified in Mascot as a fixed modification. Deamidation of asparagine and glutamine, methylation of lysine and glutamine, oxidation of methionine, and acetylation of lysine at the N terminus were specified in Mascot as variable modifications. Download FIG S3, TIF file, 1.8 MB. Copyright © 2021 Lipońska and Yap. 2021 Lipońska and Yap. https://creativecommons.org/licenses/by/4.0/ This content is distributed under the terms of the Creative Commons Attribution 4.0 International license.

### S. aureus RNase R localizes to the 30S subunit of 70S ribosomes.

**FIG 3:**
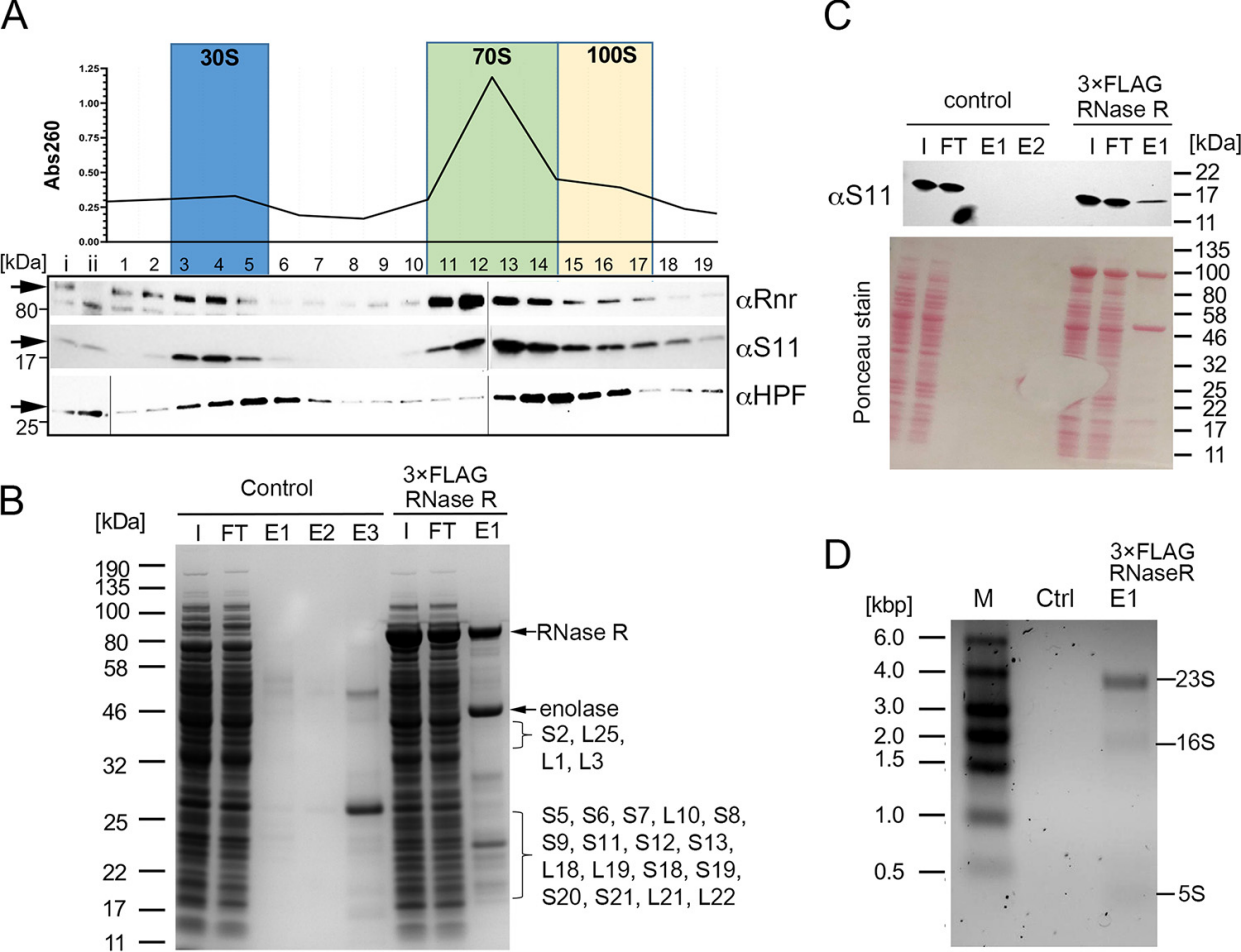
S. aureus RNase R cofractionated with the 30S and 70S ribosomes. (A) Association of RNase R and HPF with the ribosomes. RNase R is enriched in the 30S and 70S fractions. Crude ribosomes extracted from stationary-phase cultures (18 h growth at 37°C) were ultracentrifuged through a 5% to 30% sucrose density gradient (*x* axis), and each fraction was precipitated by a final concentration of 10% trichloroacetic acid (TCA), resolved on 4% to 20% SDS-PAGE, and probed with anti-HPF (1/8,000), anti-Rnr (1/1,000), and anti-S11 (1/4,000) antibodies. Total lysates from the WT (lane [i]) and Δ*rnr* (lane [ii]) strains served as references. The 30S ribosomal protein S11 served as a fractionation marker. (B) FLAG-RNase R coimmunoprecipitated proteins analyzed by EZBlue staining. Proteins copurified with FLAG-tagged RNase R and the untagged control were identified by liquid chromatography-tandem mass spectrometry (LC-MS/MS) (see Data Set S1 in the supplemental material). Both protein components of the 50S subunit and 30S subunit were overrepresented, including the S. aureus degradosome component enolase (92). I, input; FT, flowthrough; E, elution. (C) Western blot showing the enrichment of 30S ribosomal protein S11 in the eluate of the FLAG-RNase R sample obtained from experiments shown in panel B. S11 was detected in the eluate of FLAG-RNase R but not in the untagged control. (D) FLAG-RNase R co-immunoprecipitated with 23S and 16S rRNA. RNA was extracted from the affinity pulldown eluate shown in panel B, and ∼1.6 μg of RNA was analyzed on a 1% TAE denaturing agarose gel and stained with ethidium bromide.

Previous studies have shown that E. coli and S. pneumoniae RNase R can bind to the 30S subunit and 50S subunit, respectively (58–60). Sucrose gradient density fractionation and Western blots were used to determine if S. aureus RNase R is ribosome associated. We found that S. aureus RNase R predominantly cosedimented in the 30S and 70S fractions (Fig. 3A). HPF was enriched in the 30S, 70S, and 100S dimers, consistent with previous findings (11, 12). The concurrent detection of RNase R and HPF in the same fractions suggests that these proteins could bind to nonoverlapping sites of the same 30S subunit. Alternatively, it is possible that RNase R-bound and HPF-bound ribosomes represent two separate ribosome subpopulations. The association of RNase R with S. aureus ribosomes was substantiated by immunoprecipitation of FLAG-tagged RNase R and mass spectrometric identification of the pulldown products (see Data Set S1). As predicted, FLAG-RNase R selectively and specifically co-immunoprecipitated with both ribosomal proteins and rRNAs (23S and 16S) of the 70S ribosome (Fig. 3B to D). HPF was absent from the eluate, reinforcing the notion that RNase R likely interacts with ribosomes that are devoid of HPF. Critically, the same interactors were not copurified in a control input without the FLAG affinity tag (Fig. 3B to D). These findings support that S. aureus RNase R primarily binds to the 30S subunit and the 70S complex.

10.1128/mBio.00334-21.8 DATA SET S1 Proteins copurified with 3×FLAG-Rnr and identified by LC-MS/MS. Download Data Set S1, XLSX file, 0.1 MB. Copyright © 2021 Lipońska and Yap. 2021 Lipońska and Yap. https://creativecommons.org/licenses/by/4.0/ This content is distributed under the terms of the Creative Commons Attribution 4.0 International license.

### *In vitro* degradation of synthetic RNA substrate and S. aureus rRNAs.

**FIG 4:**
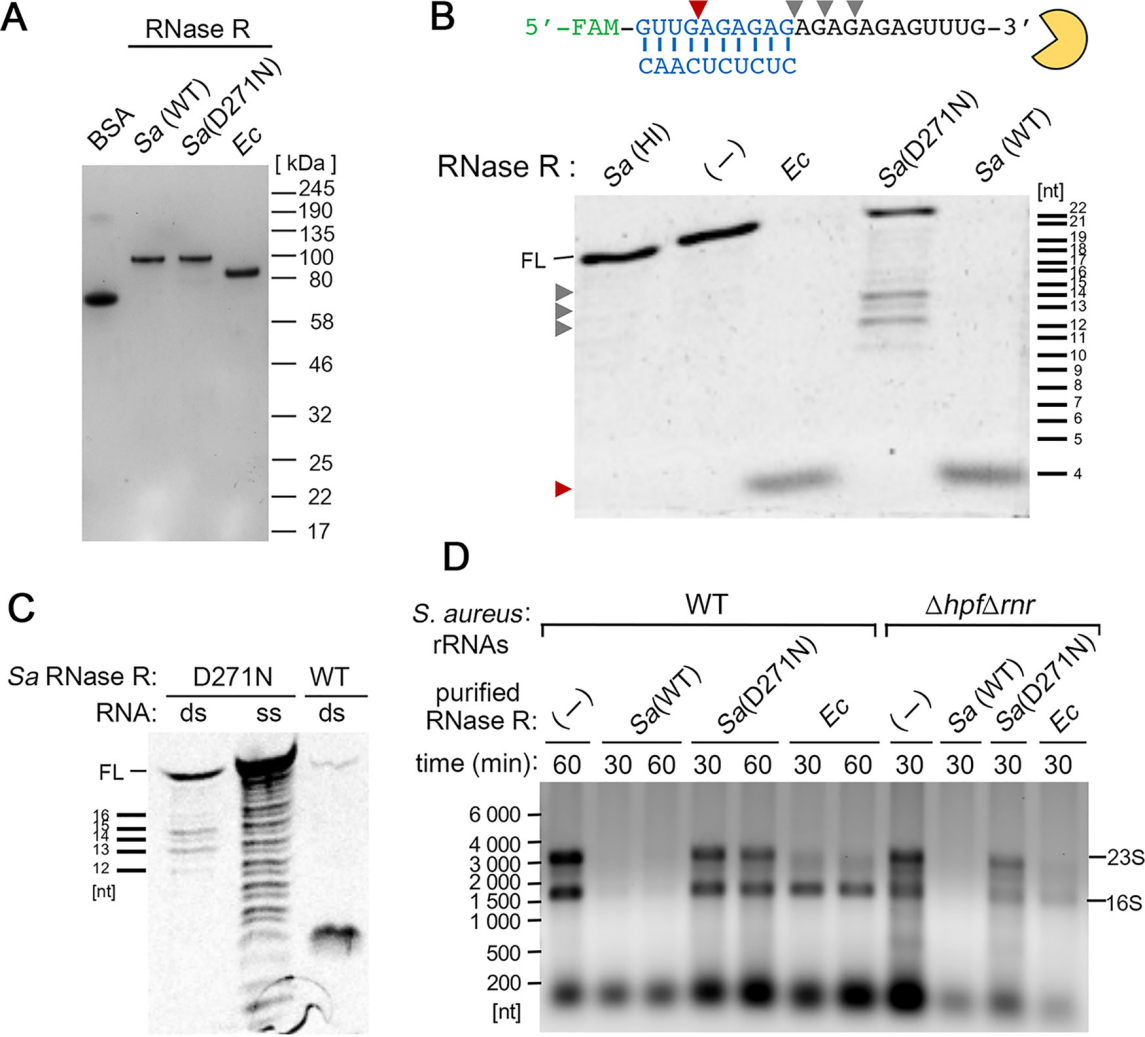
S. aureus RNase R efficiently degrades synthetic RNA duplexes and purified rRNAs *in vitro.* (A) Purification of S. aureus His_6_-RNase R [*Sa*(WT)] and its catalytically inactive RNase R [*Sa*(D271N)]. Commercially available E. coli RNase R (*Ec*) and bovine serum albumin (BSA) served as controls. Proteins were analyzed on a 4% to 20% SDS-PAGE gel and stained with EZBlue. The loading inputs were BSA (0.7 μg), *Sa*(WT) (0.18 μg), *Sa*(D271N) (0.18 μg), and *Ec* (0.36 μg). (B) Wild-type S. aureus and E. coli RNase R (55 nM each) efficiently degrade the 5′-end FAM-labeled dsRNA (2.5 μM) with a 3′ overhang, whereas RNase R (D271N) degrades only the single-stranded region. The *Sa*(WT) RNase R cleavage product is marked by a red arrow. Gray arrows indicate the cleavage sites of *Sa*(D271N). Heat-inactivated [*Sa*(HI); 95°C for 10 min) RNase R and a reaction without any enzyme (−) served as negative controls. RNA markers were generated by alkaline hydrolysis or RNase T1 digestion, which specifically cleaves after G bases. Reactions were analyzed on a 20% TBE-urea polyacrylamide gel, and fluorescence signals were visualized on an iBright FL1500 imager. FL, full-length. (C) S. aureus RNase R (D271N) retains exonuclease activity in cleaving ssRNA but is ineffective on dsRNA substrate. The same reactions were performed as that shown in panel B except that 5′-FAM-ssRNA was used (top strand in panel B). (D) Degradation of S. aureus 23S and 16S rRNA by RNase R. Wild-type S. aureus RNase R, but not its D271N variant, degrades both 23S and 16S rRNAs originating from the WT strain and the Δ*hpf* Δ*rnr* mutant, whereas the E. coli RNase R was less efficient in digesting rRNAs from S. aureus under the same conditions. In each reaction mixture, 2 μg of total RNA was incubated at 37°C for the indicated time with 55 nM RNase R enzymes. Samples were resolved on a 0.8% TAE agarose gel and stained with ethidium bromide.

The rRNAs in the S. aureus Δ*hpf* strain were significantly more degraded than those in the WT and Δ*rnr* strains (Fig. 1B). To rule out the possibility that the degradation was due to contamination during RNA isolation, we reconstituted RNase R-dependent degradation *in vitro* with cell-free purified components. An N-terminally His_6_-tagged RNase R was purified by Ni-nitrilotriacetic acid (NTA) affinity chromatography and size exclusion filtration. A catalytically dead mutant S. aureus RNase R (D271N) variant and a commercially available E. coli RNase R served as the controls throughout this study (Fig. 4A). The equivalent D280N substitution in E. coli RNase R is inactive with respect to exonuclease activity, but retains its helicase activity (61). The enzymatic activity of S. aureus RNase R was confirmed by *in vitro* degradation of a 5′-fluorescently labeled synthetic RNA duplex (Fig. 4B). The same substrate with the ^32^P-labeled 5′ end was used to examine E. coli RNase R activity (62). Both WT S. aureus and E. coli RNase R cleaved the dsRNA efficiently in the 3′-to-5′ direction, generating a 4-nt product (Fig. 4B, red arrow), whereas heat-inactivated WT S. aureus RNase R and an identical reaction without any enzyme retained the full-length substrate. Although the presumed catalytically inactive D271N variant failed to cleave the dsRNA, it was able to cleave the single-stranded 3′ overhang of a duplex (gray arrows) and degrade the single-stranded RNA (Fig. 4B and C).

RNase R homologs target all linear RNA and structured rRNAs with very little (if at all) sequence preference. Purified rRNAs are ideal substrates of RNase R because they are free of HPF and proteins to insulate them from the exonuclease. Next, total RNA was extracted from exponential-phase WT S. aureus cultures and late-stationary-phase Δ*hpf* Δ*rnr* cultures. Both S. aureus and E. coli RNase R degraded the purified rRNAs regardless of the origin of the strains, whereas the S. aureus RNase R(D271N) was inactive on the same substrates (Fig. 4D). These data biochemically confirm that the accelerated ribosome degradation observed in the Δ*hpf* strain during late stationary phase is largely attributed to the action of RNase R (Fig. 1).

### HPF-bound ribosomes are protected from S. aureus RNase R.

**FIG 5:**
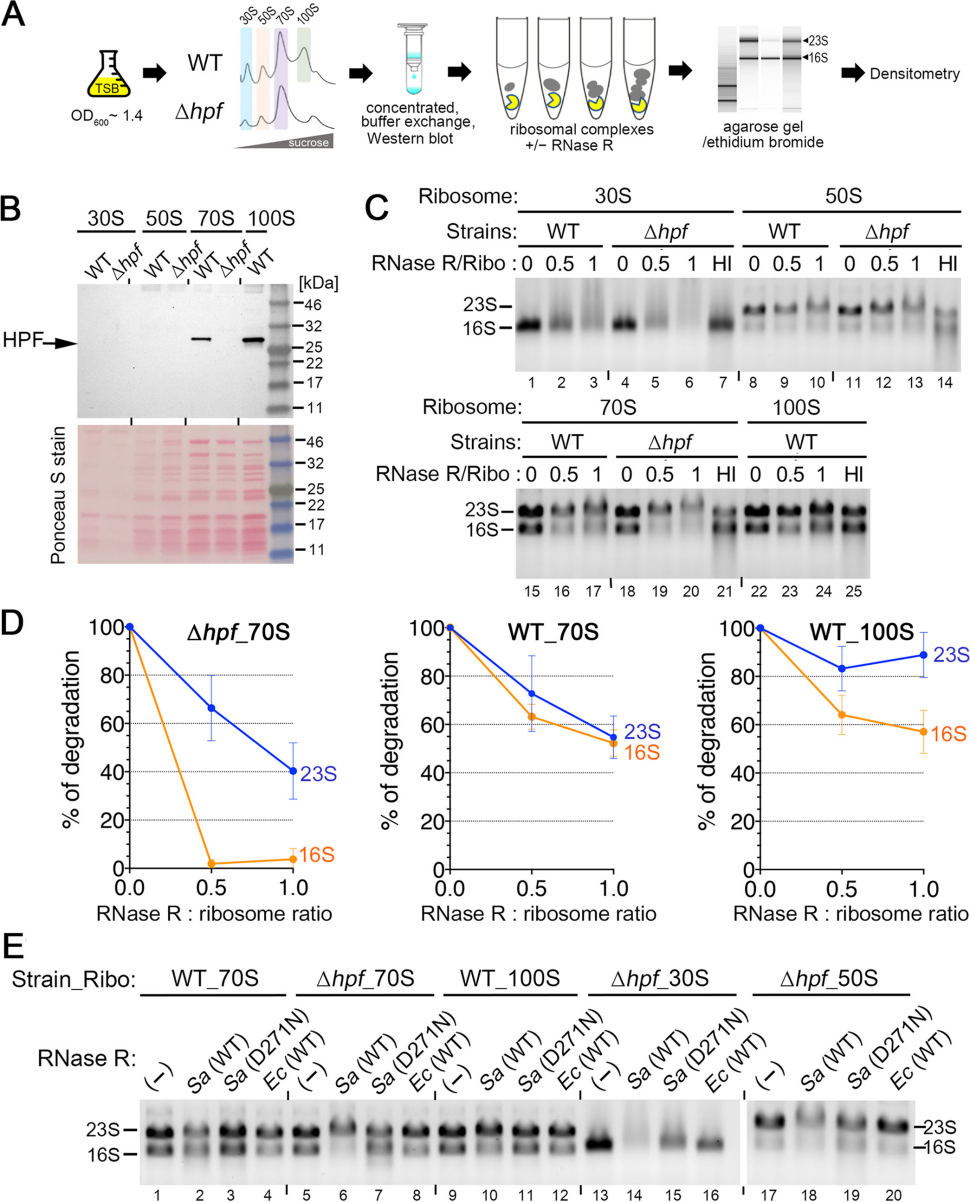
*In vitro* ribosome degradation experiments. (A) Experimental workflow of cell-free ribosome degradation. (B) Western blot confirming the association of HPF with the purified ribosomal complexes. HPF was detected in 70S and 100S ribosomes isolated from the WT strain. Each lane corresponds to 2.5 pmol of ribosomes. Ponceau S staining prior to immunoblotting (anti-HPF antibody at 1/4,000 dilution) showed the input of ribosomes from the WT and the Δ*hpf* mutant. (C) Representative 0.8% TAE denaturing agarose gel showing the degradation of 23S or 16S rRNA in the ribosomal complexes originating from the WT and Δ*hpf* strains. The 70S and 100S ribosomes from the WT were resistant to RNase R degradation. One picomole of ribosomes was used per reaction. Heat-inactivated (HI; 95°C for 10 min) RNase R reaction serves as a control. (D) Quantitation of the degrees of ribosome degradation at different RNase R-to-ribosome molar ratios. The intensities of 23S and 16S rRNA bands in panel C were quantitated by ImageJ and normalized against the no-enzyme reactions. The means ± SDs (*n* = 3) are shown. (E) Comparison of RNase R activity between WT S. aureus RNase R [*Sa*(WT)], its inactive D271N mutant [*Sa*(D271N)], and E. coli RNase R [*Ec*(WT)] on purified ribosomes. Reactions were performed at a 1:0.6 protein-to-ribosome ratio. The 30S and 70S complexes from the Δ*hpf* mutant were highly susceptible to WT S. aureus RNase R. In contrast, E. coli RNase R and S. aureus RNase R(D271N) were less active (if at all) on the 30S, 50S, and 70S ribosomes isolated from a Δ*hpf* mutant. All samples were analyzed on a 0.8% TAE denaturing agarose gel and stained with ethidium bromide. Slower migration of the 23S rRNA bands is likely due to incomplete denaturing of the RNA in the presence of recombinant RNase R.

We performed cell-free ribosome degradation assays to determine the role of HPF in ribosome turnover. Ribosomal subunits (30S and 50S), 70S ribosomes, and 100S dimers (in the case of WT) were individually isolated from the WT S. aureus and Δ*hpf* strains using sucrose density gradient ultracentrifugation (Fig. 5A). These ribosomal fractions were probed with anti-HPF to confirm the association and absence of HPF. HPF mainly localizes to the 30S subunit and 30S of a 70S complex (Fig. 3A) (27, 43–45). After ribosome purification, HPF was detected in 70S and 100S ribosomes isolated from the WT strain but not in 30S and 50S subunits (Fig. 5B). The undetectability of HPF in the 30S subunit was possibly due to the dissociation of HPF during ribosome purification. To obtain optimal stoichiometry, the ribosomes were first incubated with various molar ratios of ribosome to RNase R. The extent of degradation was quantitated by calculating the amount of remaining substrate relative to the initial input of a control without RNase R (Fig. 5D). At a 1:1 molar ratio, Δ*hpf*-derived 30S subunits were more susceptible to RNase R than those derived from the WT strain (Fig. 5C, lanes 1 to 6). The 50S subunits from both strains were equally tolerant to RNase R (Fig. 5C, lanes 8 to 14), in agreement with the 70S reactions in which 23S rRNAs were degraded less efficiently than the 16S rRNAs (Fig. 5C, lanes 17 and 20). In Δ*hpf*-derived 70S complex, 16S rRNAs were mostly degraded as opposed to partial degradation of the WT 70S, suggesting that the presence of HPF precludes RNase R action. This idea is further supported by the fact that 100S ribosomes were almost completely resistant to RNase R (Fig. 5C, lane 24; Fig. 5D).

To strengthen our observations, we repeated the ribosome degradation experiments and compared the RNase activity of WT S. aureus RNase R, its D271N mutant, and E. coli RNase R. Consistent with our previous experiments, we found that 100S ribosomes are more tolerant than 70S ribosomes, implying that 70S dimerization enhances RNase R resistance. In contrast, 16S rRNAs in the Δ*hpf-*generated 30S subunits and Δ*hpf*-generated 70S ribosomes were fully degraded by the S. aureus RNase R (Fig. 5E, lanes 6 and 14). Surprisingly, E. coli RNase R did not cleave S. aureus ribosomes as efficiently as we originally anticipated (Fig. 5E, lanes 4, 8, 12, and 16) despite exhibiting comparable nucleolytic activity on synthetic substrates and purified rRNAs (Fig. 4) under the same degradation conditions previously used for testing E. coli RNase R activity (62, 63). The difference could be due in part to species-specific features of the bacterial ribosomes (64) that indirectly affect RNase R binding and catalysis or simply due to biochemical differences between S. aureus and E. coli RNase R, which only share 37% protein sequence identity (58% similarity).

### S. aureus 16S rRNA is cleaved at specific sites in the absence of HPF.

**FIG 6:**
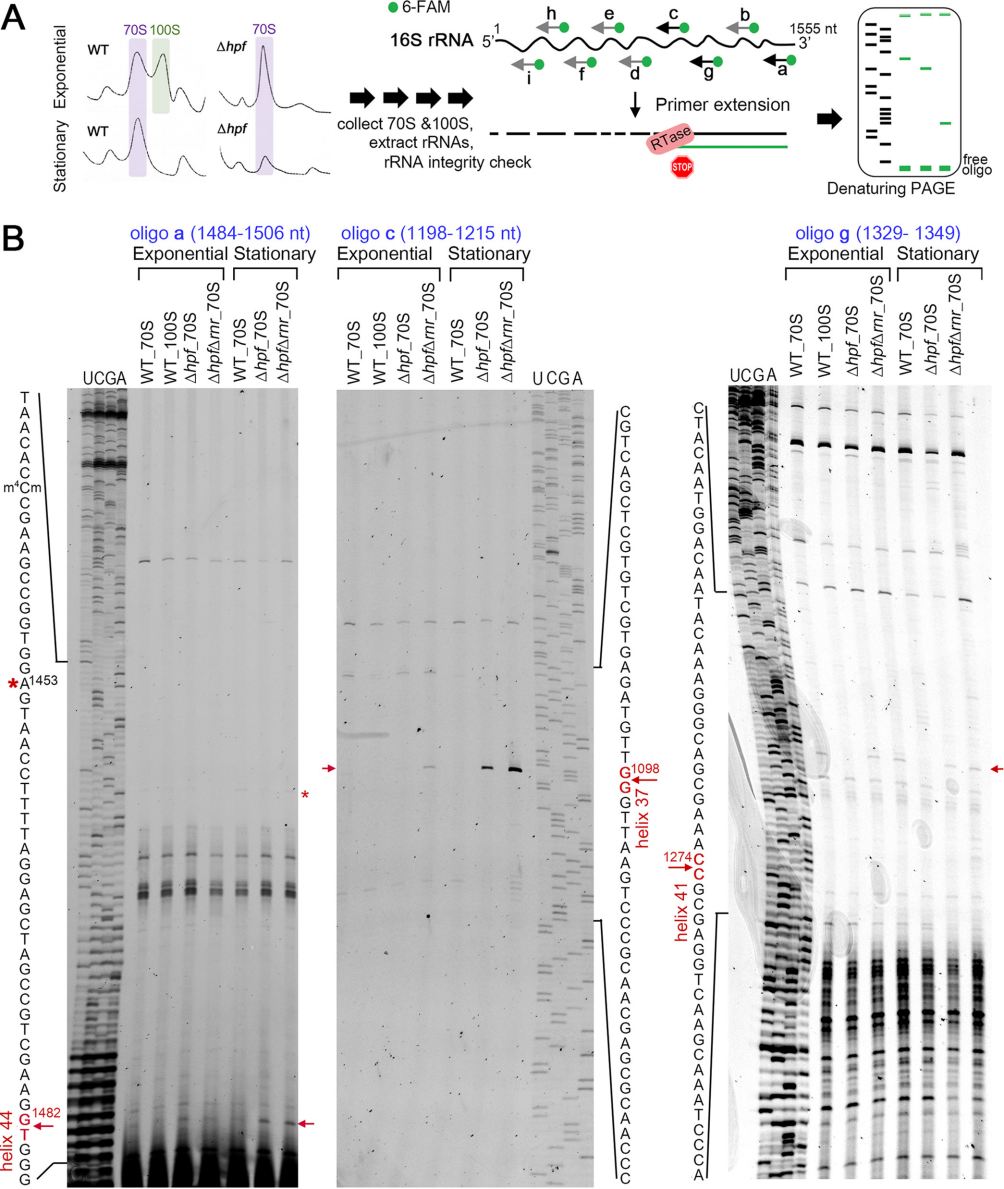
Mapping the HPF-protected 16S rRNA regions by primer extension. (A) Schematic of fluorescence-based primer extension. Reverse transcriptase (RTase) halts at 5′ end of a cleaved RNA template and generates a truncated cDNA that is subsequently analyzed on a 6% (oligonucleotide “c”) or 10% (oligonucleotides “a” and “g”) TBE-urea polyacrylamide gel. 6-FAM, 6-carboxyfluorescein. (B) Primer extension analysis using oligonucleotides a, c, and g to detect differentially cleaved rRNAs within h44, h37, and h41, respectively. The red arrows indicate the position of a cleavage product. An asterisk indicates a stationary-phase-specific intermediate. “UCGA” marks the sequencing ladder; also see Fig. S4.

To determine the regions that are protected in the HPF-ribosome complexes, we performed primer extension mapping to identify the 5′ RNA ends of degraded intermediates using total rRNA extracted from individually isolated 70S and 100S ribosomes (Fig. 6A). A total of nine fluorescently labeled antisense oligonucleotides (a to i) were used for primer extension to cover >90% of the 1,555-nt long S. aureus 16S rRNA. Significant changes in 16S rRNA cleavage patterns between WT- and Δ*hpf*-derived ribosomes were only observed with primers a, c, and g (Fig. 6B). Reverse transcription reactions using primer “a” identified a prominent 16S rRNA intermediate within the h44 with the cleavage site at G1482 (E. coli numbering U1471) that was predominantly present in the 70S ribosomes isolated from Δ*hpf* backgrounds, particularly during stationary phase. An additional cut site at A1453 occurred during stationary phase and was detected in WT 70S ribosomes, but the same band was also weakly visible in the Δ*hpf* 70S ribosome (Fig. 6B, left, *). Using primer “c,” a strong intermediate corresponding to a cleavage site in h37 after G1098 (E. coli numbering G1089) was exclusively detected in the 70S ribosome obtained from Δ*hpf* backgrounds (Fig. 6B, middle). The third differentially cleaved fragment obtained from primer “g” was cut within h41 after C1274 (E. coli numbering U1264). This intermediate was found in Δ*hpf* 70S ribosomes isolated from both exponential- and stationary-phase cells but not in the 100S ribosomes (Fig. 6B, right). That the cleaved species from primer” c” accumulated in the Δ*hpf* Δ*rnr* double mutant in both growth phases indicates that the intermediate was derived from RNase R action. In contrast, no significant accumulation of fragments from primers “a” and “g” was observed in the double mutant, supporting our previous notion that an unidentified RNase is involved (Fig. 1). The precise locations of the three cleavage sites in the S. aureus 16S rRNA are presented in Fig. S4. These results indicate that 70S ribosomes without HPF are exposed to nucleolytic cleavage at distinct regions from the HPF-harboring WT 70S and 100S complexes.

10.1128/mBio.00334-21.7 FIG S4 Secondary structure of 16S rRNA from S. aureus USA300_FPR3757. Image is adapted from RNAcentral database (https://rnacentral.org/rna/URS00001B1A25/451515) (91). The red scissors indicate the cleavage sites detected in the Δ*hpf* strain. aSD, anti-Shine-Dalgarno FIG S4, TIF file, 0.5 MB. Copyright © 2021 Lipońska and Yap. 2021 Lipońska and Yap. https://creativecommons.org/licenses/by/4.0/ This content is distributed under the terms of the Creative Commons Attribution 4.0 International license.

**FIG 7:**
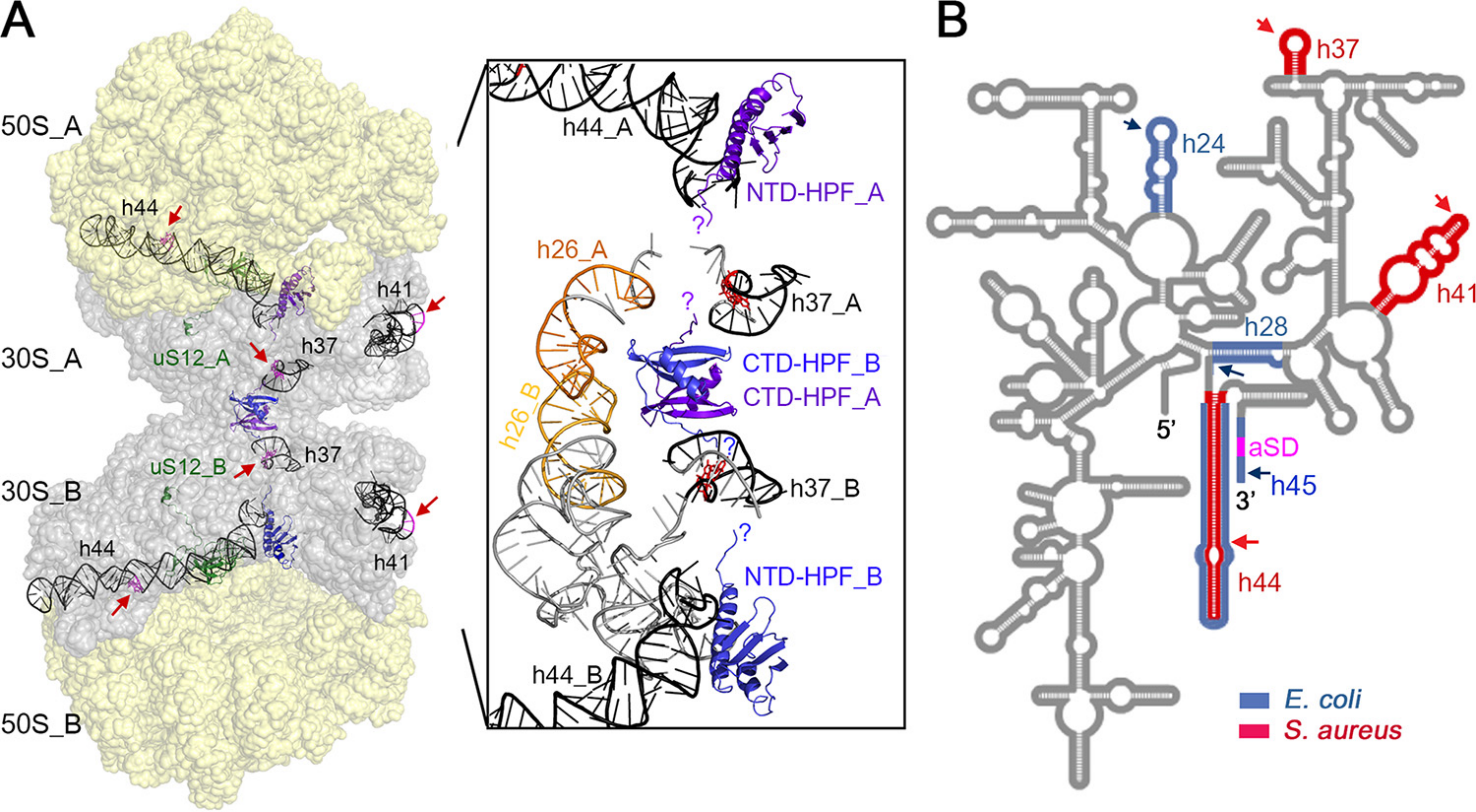
Comparison of hibernation factor-protected sites. (A) Locations of h37, h41, and h44 on the S. aureus 100S ribosome (PDB 6FXC). The two 70S monomers form an interface via the 30S subunits (gray) and are tethered together by two CTD-HPF molecules (purple blue and marine blue). For simplicity, a secondary interaction between uS2 and h26 is omitted. Cleavage sites are indicated by red arrows and highlighted in magenta. (Inset) A closeup view of the 30S-30S dimerization interface. Most rRNA helices have been manually removed to better show the interactions of the two CTD-HPF molecules. NTD-HPF interacts with several rRNA helices, including h44. The termini of the 35-aa unstructured HPF linker are indicated with question marks. A potential RNase R interactional partner, uS12, is marked in green. CTD, C-terminal domain; NTD, N-terminal domain. (B) A diagram of bacterial 16S rRNA secondary structure showing the relative locations of hibernation factor-protected sites. rRNA helices that are susceptible to nucleolytic degradation are marked in red (S. aureus) and blue (E. coli). E. coli cleavage sites are deduced from reference 67. aSD, anti-Shine-Dalgarno.

Positioning the differentially cleaved sites to a cryo-electron microscopy (cryo-EM) structure of S. aureus 100S ribosome (PDB 6FXC) revealed that they are located at the critical components of the ribosome (Fig. 7A). Although the observed cleavage site at G1482 (E. coli U1471) of h44 resides in the variable region of B6R bridge formed between h44 and L19, fragmentation of h44 could potentially collapse several intersubunit bridges (B2a/d, B3, B5, B6, and B6R) (65). The rRNA in the individual 30S and 50S subunits at the subunit interface are mostly solvent exposed. The binding of NTD-HPF to the distal end of h44 in the 70S ribosome may prevent accessibility of RNase. h35-h37 interacts with S2 within the head of 30S subunit, forming part of the mRNA channel that accommodates the Shine-Dalgarno duplex. h37 is potentially on the path of the unresolved 35-amino-acid (aa) flexible linker that connects the two HPF domains. Perturbations within the linker are known to completely abolish 70S dimerization function (10, 27). It is possible that this unstructured linker plays a role in RNase occlusion. h41 is surface exposed and may be intrinsically susceptible to RNase. Curiously, E. coli h41 binds to RNase I to inhibit its nucleolytic activity (66). Recently, the RNase sensitive rRNA regions (h24, h28, and h44 to h45) have been mapped in E. coli cells lacking all three hibernation factors (RMF, HPF, and YfiA) (67). Comparing the E. coli and S. aureus 16S rRNA cleavage profiles clearly demonstrates that hibernation factor(s) from these bacteria protects different regions of the ribosomes (Fig. 7B).

## DISCUSSION

**FIG 8:**
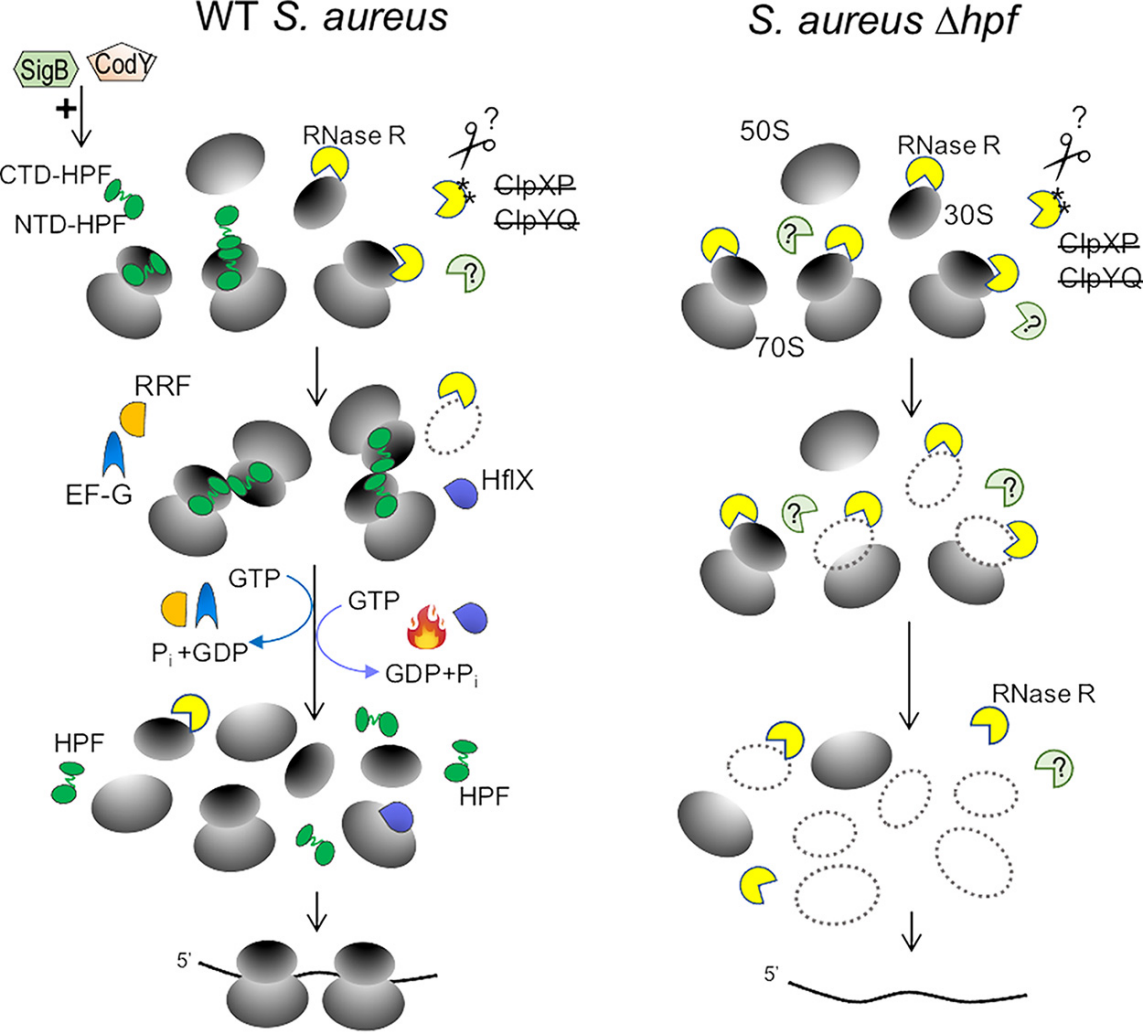
A model for the protective role of 70S dimerization against ribosome degradation. (A) In WT S. aureus, the expression of *hpf* is positively regulated by CodY and SigB transcription factors (22). The N-terminal domain of HPF (NTD-HPF) binds to the decoding regions of the 30S subunit of a 70S ribosome, blocking the mRNA binding and entry of tRNAs and inactivating translation. The C-terminal domain of HPF (CTD-HPF) mediates 70S dimerization by directly interacting with the CTD-HPF of the opposite copy of CTD-HPF on another 70S monomer, joining the two 70S monomers in a side-to-side configuration to form the hibernating 100S ribosomes. The HPF-bound 70S and HPF-bound 100S complexes are resistant to RNase R-dependent degradation during the stationary phase. S. aureus RNase R is methylated at Q538, and Q537 structurally mimics an acetyl lysine (labeled with asterisks). Inactivating the major ClpYQ and ClpXP proteases does not enhance the protein stability of RNase R. The scissors mark an unknown protease. When conditions become favorable, 100S ribosomes are disassembled into recyclable ribosomal complexes by the RRF/EF-G pair or via the HflX-dependent pathway under heat stress. Both processes require GTP hydrolysis to split the subunits. The disassembly of 100S ribosomes allows rapid reinitiation of translation, bypassing the energy-consuming ribosome biogenesis steps. (B) In a Δ*hpf* knockout, all 30S and 70S ribosomal complexes are exposed to RNase R and other RNases (light green pies with question marks), resulting in a total loss of ribosome pools and eventual cell death under nutrient stress.

A causative link between 70S dimerization and ribosome turnover has only emerged recently. Here, we report that the binding of S. aureus HPF to a 70S monomer provides some protection against RNase R-mediated degradation, and 70S dimerization (a 100S complex) provides the greatest protection. The free 30S subunits are susceptible to RNase R, presumably due to the low-affinity binding of HPF. The 50S subunits are intrinsically tolerant to RNase R regardless of the genetic background (WT versus Δ*hpf* mutant) because 50S is not the native interactional partner of RNase R. Unlike E. coli RNase R, S. aureus RNase R turnover is not modulated by lysine acetylation and ClpYQ (HslUV) protease. We propose a model by which hibernating ribosomes (HPF-bound 70S and 100S) serve as a reservoir to preserve unused ribosomes. When a demand for translation increases, e.g., upon exit from dormancy and in response to a specific stressor, ribosome hibernation is reversed via dissociation and recycling of ribosomes split by either the RRF/EF-G disassembly pathway (24) or the heat-induced HflX-mediated pathway (25), allowing initiation of new translation (Fig. 8A). In contrast, all ribosomes in the Δ*hpf* strain are vulnerable to RNase R and other RNases, resulting in almost complete loss of ribosomes and inability to resume new translation (Fig. 8B), consequently leading to cell death during prolonged nutrient deprivation (27) and loss of virulence (22). S. aureus RNase R is expressed in log phase when the HPF concentration is lower than in stationary phase (Fig. 2). We posit that active translation at exponential phase renders cells resistant to RNase R. During exponential growth, the majority of ribosomes are actively engaged in translation, and it is possible that a low level of HPF is stoichiometrically sufficient to protect a smaller fraction of idle ribosomes. Translating ribosomes are either not or a poor substrate of RNase, because elongating ribosomes are conformationally dynamic and constantly associate with translation factors that may block or compete with the RNase. Furthermore, RNase-sensitive 30S-50S intersubunit regions are not solvent exposed in the translating ribosomes. Alternatively, RNase R may be kept inactive in log phase by an unknown binding partner.

Remarkably, while S. aureus HPF protects h37, h41, and h44 of the 16S rRNA (Fig. 7; see also Fig. S4 in the supplemental material), the three E. coli hibernation factors block the nucleolytic cleavage at distinct regions (h24, h28, and h44 to h45) (67). These differences could be due in part to species-specific variations in rRNA helices (extensions, deletions, and alternative fold) (68) and disparate interactions of E. coli RMF and S. aureus CTD-HPF with the 30S subunit (11, 12, 23). E. coli YbeY (a single-strand specific endoribonuclease) is critical in processing 17S precursor and, together with RNase R, is known to degrade nonfunctional 70S and 30S complexes (50). E. coli YbeY and RNase R were reported to concertedly degrade 16S rRNA in a mutant lacking all three hibernation factors (67). It is unclear whether YbeY and RNase R can directly act on assembled ribosomes, as the experiments were performed with total cellular RNA. In S. aureus, a Δ*ybeY* mutant is defective in ribosome biogenesis, leading to a significant reduction of 100S dimer formation (Fig. S1). When combined with Δ*hpf* deletion, the Δ*hpf* Δ*ybeY* double mutant exhibits severe growth defects, accumulates 50S subunits, and fails to slow ribosome loss (Fig. S1), suggesting that YbeY is unlikely to participate in ribosome degradation in a S. aureus Δ*hpf*. That inactivation of *rnr* does not completely rescue the ribosome content to the WT level (Fig. 1A) strongly suggests that an unidentified endoribonuclease is involved in generating the substrates for RNase R. This conjecture is supported by the observation of growth impairment in the Δ*hpf* Δ*rnr* double mutant, in which HPF-free ribosomes that are preserved upon RNase R removal could presumably still be targeted by the unknown RNase, generating partially damaged and not fully active ribosomes. Inactivation of *rnr* could lead to accumulation of toxic rRNA precursors and damaged tRNAs, these toxicities, together with the partially active ribosomes, may exacerbate translational capacity and lead to growth defect.

The precise mechanism of HPF-mediated protection remains to be investigated. It is conceivable that HPF directly competes for the binding of RNase R or indirectly reduces RNase R-30S association and nuclease activity. Alternatively, 70S dimerization may hinder the accessibility of RNase R adjacent to the 30S-30S dimerization interface. E. coli RNase R directly interacts with uS12 (69) that forms a part of the B2a bridge. The NTD-uS12 is anchored in the interior of 30S while CTD-uS12 lies on the surface (Fig. 7A), it is possible that uS12 serves as the docking site for RNase R. An attempt to model a truncated E. coli RNase R (PDB 5XGU) to multiple 70S ribosome structures and the S. aureus 100S complex (PDB 6FXC) was unsuccessful. Chemical footprinting may offer some insights into the ribosome binding sites of RNase R, but due to its size (∼92 kDa), which potentially occupies a large area, it is very likely that the results will be inconclusive. Solving the cryo-EM structures of various ribosome-RNase R cocomplexes is an ongoing effort but extends beyond the scope of this study.

RNase R (formerly VacB) is required for pathogenesis in Campylobacter jejuni, Shigella flexneri, E. coli, Brucella abortus, Aeromonas hydrophila, Helicobacter pylori, and Legionella pneumophila (70–75). Only E. coli RNase R has been extensively studied. E. coli carries an RNase R paralog, RNase II, that is also involved in rRNA decay, but RNase II is absent in S. aureus (76). Bacterial RNase R is a multifunctional nonspecific 3′-5′ exonuclease. It has been shown to remove aberrant rRNAs and to cleave repetitive extragenic palindromic (REP) elements (77), to act concertedly with RNase II or YbeY to degrade defective ribosomes, to coordinate with Hfq and PNPase for RNA quality control, to process structured RNAs (tmRNA, tRNA, and rRNA), and to destroy nonstop mRNAs in a stalled ribosome (49–52, 78). Very limited mRNAs were identified in a recent S. pyogenes RNase R targetome study (79), implying that intact mRNAs are not the primary targets of RNase R. Many binding partners of E. coli RNase R have also been identified, including the tmRNA-SmpB complex that recruits RNase R to the elongation-arrested ribosomes, sRNA binding Hfq (78), and β-methylthioaspartic acid-modified uS12 that promotes RNase R binding (69). With the exception of uS12, none of the aforementioned interactors (including YbeY and PNPase) co-immunoprecipitated with the S. aureus RNase R (Fig. 3B; Data Set S1). The data suggest either that these factors target distinct pools of ribosomes or that the factors were not expressed at sufficient levels (or were sequestered) under the tested conditions.

It is unclear whether the repertoire of E. coli RNase R targets and interactors are applicable to S. aureus, given some major mechanistic differences and key players involved in RNA metabolism between Gram-negative and Gram-positive bacteria (48, 49, 51, 52). Likewise, differences may occur at the level of regulation. For instance, Pat(YfiQ/Pka)-acetylated E. coli RNase R is degraded by ClpYQ (HslUV) and Lon proteases during the exponential phase. The stability of E. coli RNase R increases upon entry into stationary phase concomitantly with a downshift of Pat (YfiQ/Pka) abundance (55, 80). S. aureus RNase R is not acetylated at the equivalent position (K544 in E. coli versus Q537 in S. aureus). In fact, K544 is not universally conserved among bacterial homologs, and in S. aureus, it is replaced by the acetylated lysine-mimic glutamine (Fig. S3). Furthermore, the homolog of Pat (YfiQ/Pka) and Lon protease are absent in S. aureus. Our mass spectrometry analyses show that Q538 of S. aureus RNase R is methylated (Fig. S3). Future studies are needed to dissect how Q538 methylation affects RNase and helicase activities, protein-ribosome and protein-rRNA interactions, and RNase R stability.

RNase R homologs are evolutionarily conserved from bacteria to humans. The human homologs Dis3L and Dis3L1 are part of the exosome components participating in RNA processing and degradation. The third homolog, Dis3L2, is involved in the processing of microRNAs (miRNAs) and noncoding RNAs (ncRNAs). A loss of function of Dis3L proteins is linked to many human diseases (62, 81). Hibernating 80S ribosomes are relatively stable (6, 7, 19), and it remains to be determined whether they are protected by preclusion of the action of Dis3L exonucleases and known ribosome degradation pathways such as the ubiquitin-proteasome system and ribophagy (82). Our discovery fills the knowledge gap between ribosome hibernation and turnover and may delineate general principles of RNase R function in all kingdoms of life.

## MATERIALS AND METHODS

### Strains, plasmids, chemicals, and growth conditions.

Strain JE2 is a community-associated methicillin-resistant Staphylococcus aureus (CA-MRSA) of USA300 lineage (GenBank CP000255) (83). The JE2 RNase mutant derivatives carry a *bursa aurealis* transposon insertion were acquired from BEI Resources (see Table S1 in the supplemental material).

The in-frame *hpf* deletion mutant (strain MNY133) was constructed as follows: a 2-kb flanking region of the *hpf* (locus SAUSA300_0736) was PCR amplified with the primer pairs P0687/0688 and P0689/P0690 via 2-step PCR using S. aureus JE2 genomic DNA as the template. The product was digested with SalI and SacI and cloned into the same sites of pBT2 (84). The resulting pBT2 Δ*hpf* was digested with SmaI, dephosphorylated, and ligated to the blunt-ended ∼1.6-kb kanamycin (Km) resistance cassette that was released from pBTK (84) by KpnI and HindIII digestion. The resulting construct pBT2 Δ*hpf*::Km was passaged through S. aureus RN4220 or E. coli DC10B, and the plasmid was reisolated, electroporated into S. aureus JE2, and selected at 30°C on agar plates supplemented with 10 μg/ml chloramphenicol. The integrant was furthered selected by a 43°C temperature upshift on chloramphenicol-containing agar plates. The homologous recombinant was resolved by 30°C passages and cycloserine enrichment according to the published procedures (84). Seventy-five micrograms per milliliter of kanamycin was used for recombinant selection. The Δ*ybeY*::Erm allele (*ybeY* locus, SAUSA300_1530) was constructed with the same strategy, except that primer pairs P1247/P1238 and P1239/P1240 and a 1.3-kb erythromycin resistance marker (from pBTE) were used. The RNase transposon mutant alleles (Table S1) were subsequently transferred to the isogenic MNY133 to create the double mutants via Φ11 phage transduction.

To overexpress 6×His-Rnr, primers P1436/P1437 were used to amplified ∼2.4-kb *rnr* using JE2 DNA as a template and cloned into an isopropyl-β-d-thiogalactopyranoside (IPTG)-inducible pMCSG7 via a ligation independent approach (85). To create a xylose-inducible 3×FLAG-Rnr, P1462/P1463 primer pair were used to amplified ∼2.4-kb *rnr* from JE2 genomic DNA and cloned into the EcoRI and KpnI sites of pEPSA5 (86). Primers P1464/P1465 were used to introduce D271N substitution in the RNase R using a site-directed QuikChange mutagenesis kit (Agilent Genomics). Primers and RNA oligonucleotides were purchased from IDT DNA and are listed in Tables S2 and S3.

10.1128/mBio.00334-21.2 TABLE S2 Primers used in this study. Download Table S2, DOCX file, 0.1 MB. Copyright © 2021 Lipońska and Yap. 2021 Lipońska and Yap. https://creativecommons.org/licenses/by/4.0/ This content is distributed under the terms of the Creative Commons Attribution 4.0 International license.

10.1128/mBio.00334-21.3 TABLE S3 6-Carboxyfluorescein (FAM)-labeled or unlabeled RNA and DNA oligonucleotides used in this study. Download Table S3, DOCX file, 0.1 MB. Copyright © 2021 Lipońska and Yap. 2021 Lipońska and Yap. https://creativecommons.org/licenses/by/4.0/ This content is distributed under the terms of the Creative Commons Attribution 4.0 International license.

Unless otherwise noted, S. aureus cells were grown at 37°C in tryptic soy broth (TSB; Difco) at a 5:1 tube- or flask-to-medium ratio with a 1:100 dilution of an overnight seed culture. E. coli cells were grown in LB (Difco). Cold shock was performed by growing S. aureus at 37°C until an optical density at 600 nm (OD_600_) of 0.4 to 0.5 in TSB and transferred to a 16°C incubator shaker. Ten milliliters of culture was collected every hour for 2 h for downstream immunoblot analyses. When necessary, erythromycin, chloramphenicol, kanamycin, ampicillin, xylose, and IPTG were used at 5 μg/ml, 10 μg/ml, 75 μg/ml, 100 μg/ml, 12 mM, and 0.5 mM, respectively. All chemicals were from Sigma-Aldrich unless otherwise noted.

### Ribosome sedimentation profiles.

Crude ribosomes were isolated from S. aureus by cryo-milling methods in buffer A (20 mM HEPES [pH 7.5], 14 mM magnesium acetate [MgOAc_2_], 100 mM KCl, 0.5 mM phenylmethylsulfonyl fluoride [PMSF], 1 mM dithiothreitol [DTT]) (27, 43). Five absorbance units (*A*_260_) of ribosomes were layered on a 5% to 30% sucrose gradient that was prepared on a BioComp Gradient Master. The samples were centrifuged at 210,000 × *g* at 4°C in a SW41 rotor in a Beckman Coulter Optima XPN-100 ultracentrifuge for 3 h. Fractionation was performed using a Brandel fractionation system equipped with a UA-6 UV detector. To quantitate the abundance of total ribosome particles relative to that of the single Δ*hpf* mutant, the boundaries of ribosomal peaks were manually selected from the trough between the peaks. The total area under a peak was calculated by ImageJ and divided to obtain the ratio. When immunoblotting was needed, ∼200 μl per fraction was collected and subjected to final 10% trichloroacetic acid precipitation. The pellets were washed with cold acetone once, resuspended in 50 mM Tris base containing Laemmli sample buffer, and resolved by 4% to 20% TGX SDS-PAGE (Bio-Rad).

### Measurements of bacterial doubling time.

S. aureus cells were grown at 37°C in TSB. A minimum of 6 optical density measurements were taken between an OD_600_ of 0.1 to 0.9. The log_10_ of the OD_600_ values was plotted in Microsoft Excel as a function of time (in minutes). Linear regression was used to estimate the slope (m) of a curve. The doubling time was evaluated as log_2_/m.

### Total RNA purification.

S. aureus total RNA was extracted using a modified hot-phenol-SDS method (87, 88). Briefly, 10 ml of TSB cultures was centrifuged and washed twice with 1× volume of cold killing buffer (20 mM Tris-HCl [pH 7.5], 5 mM MgCl_2_, 20 mM NaN_3_). Cells were resuspended in 1 ml protoplast buffer (25% [wt/vol] sucrose, 50 mM Tris-HCl [pH 8], 0.25 mM EDTA, 50 μg/ml lysostaphin) and incubated on ice for 10 to 20 min. Protoplasts were collected at 4°C, 20,000 × *g* for 2 to 5 min. After suspension in 1 ml T_10_E_1_, the samples were added to 0.5× volume of boiled lysis buffer (200 mM NaCl, 2% SDS, 16 mM EDTA). Lysis of protoplasts was achieved by heating the cell suspension at 95°C for 10 min. The samples were extracted 3 times with acid phenol-chloroform (pH 4.5; Amresco) and once with chloroform-isoamyl alcohol (24:1; Amresco). The final aqueous phase was precipitated with 1× volume of isopropanol and one-tenth volume of 3 M sodium acetate (NaOAc) (pH 5.2; Alfa Aesar), and final RNA pellets were washed once with 70% ethanol. RNA integrity was analyzed on a 0.8 to 1% Tris-acetate-EDTA (TAE) denaturing agarose gel and stained with ethidium bromide. RiboRuler high range RNA ladder (Thermo Fisher number SM1821) was used to estimate RNA size.

### Antibodies and Western Blots.

S. aureus cell pellets were homogenized with lysing matrix B (MP Biomedicals; 100 mg beads/ml cells) in 25 mM Tris (pH 7.5) on a Retsch MM400 mixer mill at 15 Hz in four 3-min cycles. Clarified lysates were recovered by spinning at 20,817 × *g* at room temperature for 5 min to remove cell debris. A total of 0.1 to 0.2 *A*_280_ units of cell lysate were analyzed on 4% to 20% TGX SDS-PAGE gels (Bio-Rad), and the proteins were transferred to a nitrocellulose membrane using a Trans-Blot Turbo system (Bio-Rad). The membrane was stained with Ponceau red (Amresco) to ensure equal loading, followed by immunoblotting using anti-Rnr (1/1,000 dilution), anti-S11 (1/4,000 dilution), anti-HPF (1/4,000 to 1/8,000 dilutions), and anti-FLAG (1/1,000) antibodies. Polyclonal rabbit anti-S11 (25) and anti-HPF (43) antibodies were generated and described previously. Anti-FLAG M2 was from Cell Signaling (catalog number 2368). To generate anti-Rnr antibody, two peptides corresponding to residues (234 to 257 and 576 to 595) of the S. aureus RNase R (Cys-^234^QEAEAVPDHIENTEIKGRHDLRDE^257^ and Cys-^576^RKYLIEKSMDNKEVKRWEDK^595^, respectively) were custom synthesized and used for immunization in New Zealand white rabbits (Pacific Immunology). Horseradish peroxidase (HRP)-conjugated protein A (1/15,000 dilution) was from Cytiva (catalog number NA9120).

### RNase R overexpression and purification.

The overexpression and purification of the His-tagged recombinant proteins using Ni-NTA affinity chromatography have been described in detail previously (25, 27). Selected fractions of purified His-tagged Rnr were loaded on a high-molecular-weight-cutoff Amicon Ultra centrifugal filter unit (MWCO-100; Millipore) to concentrate the proteins in buffer B (40 mM HEPES [pH 7.5], 0.5 M KCl, 10% glycerol). E. coli RNase R was purchased from Lucigen (catalog number RNR07250).

### FLAG affinity pulldown and mass spectrometric analyses.

S. aureus carrying the pEPSA5 derivatives (Table S1) were grown in 200 ml TSB supplemented with 10 μg/ml chloramphenicol at 37°C to an OD_600_ of 0.4 to 0.5. A final 12 mM xylose was added to induce the expression of 3×FLAG-Rnr for 22 to 24 h. Cells were disrupted using cryo-milling method in buffer C (50 mM Tris-Cl [pH 7.5], 100 mM KCl, 1 mM PMSF, 10 mM MgCl_2_). Approximately 5 ml of cell lysates was incubated with 200 μl anti-FLAG M2 magnetic beads (Sigma-Aldrich, M8823-5ML) at room temperature (∼22°C) on a tube rotator for 1 h and an additional 15 h at 4°C. The magnetic beads were washed extensively with buffer C (7 × 1 ml) and proteins were eluted with 100 μl glycine (pH 2.6). Samples were neutralized and analyzed on a 4% to 12% Bis-Tris NuPAGE gel (Invitrogen). Liquid chromatography-tandem mass spectrometry (LC-MS/MS) was performed by the Northwestern University Proteomics Core to identify protein species and posttranslational modification after trypsin digestion. Scaffold (version Scaffold_4.11.0; Proteome Software Inc., Portland, OR) was used to validate MS/MS-based peptide and protein identifications. Peptide identifications were accepted if they could be established at greater than 99.0% probability by the Peptide Prophet algorithm with Scaffold delta-mass correction. Protein identifications were accepted if they could be established at greater than 99.0% probability to achieve a false-discovery rate (FDR) of less than 1.0% and contained at least 4 identified peptides.

### *In vitro* degradation of synthetic RNA, rRNA, and ribosomal complexes.

Reactions with 5′-6-carboxyfluorescein (FAM)-labeled RNA were carried out with 2.5 μM dsRNA or single-stranded RNA (ssRNA) (Table S3) in a total volume of 10 μl containing 20 mM Tris-HCl (pH 7.5), 100 mM KCl, 0.25 mM MgCl_2_, and 55 nM purified RNase R. Control reactions were performed in either buffer B or heat-inactivated RNase R (at 95°C for 10 min). dsRNA substrates were prepared by annealing RNA1 and RNA2 in buffer D (300 mM KCl, 30 mM Tris [pH 7.5], 1 mM MgCl_2_) for 2 min at 95°C and slowly cooled down to 25°C (at 1°C/25 s). Cleavage reaction mixtures were incubated at 37°C for 30 min and stopped by the addition of 10 μl 2× SEQ loading dye (0.05% bromophenol blue, 0.05% xylene cyanol FF, 20 mM EDTA [pH 8.0] and 91% formamide). Samples were analyzed on a 20% Tris-borate-EDTA (TBE)-urea polyacrylamide gel at a constant 180 V for 70 min. Two RNA markers were used: microRNA marker (New England BioLabs, N2102S) and 2.5 μM FAM-RNA1 were hydrolyzed at 90°C for 5 min in alkaline buffer containing 0.5 M Na_2_CO_3_ and 10 mM EDTA. The same reactions were performed for rRNA degradation except that 2 μg of total RNA were used per reaction. For ribosome degradation, 1 pmol of ribosome was programmed with and without purified RNase R at molar ratios of 0, 0.5, and 1. The rRNA- and ribosome-containing reactions were analyzed on a 1% TAE agarose gel and stained with ethidium bromide.

### Purification of ribosomal complexes.

Twenty-five absorbance units (*A*_260_) of crude ribosomes were layered on a 5% to 30% sucrose gradient and fractionated as described in “Ribosome sedimentation profiles.” The 30S, 50S, 70S, and 100S peaks were collected, buffer exchanged, and concentrated on Amicon Ultra centrifugal filter units (MWCO 100 kDa, Millipore) in buffer A. Ribosomes were quantified according to the *A*_260_ value (1 *A*_260_ = 23 pmol/ml 70S) (89).

### Mapping rRNA cleavage sites by primer extension.

Ribosomal complexes were fractionated by sucrose density ultracentrifugation as described above. The 70S and 100S peaks were collected and subjected to acidic phenol-chloroform extractions, and rRNAs were precipitated by isopropanol. Two hundred fifty nanograms of total rRNA was used for primer extension as described previously (90) using the 5′-end fluorescently labeled antisense oligonucleotides (Table S3). DNA sequencing ladders were generated using a USB Thermo SEQ kit (Affymetrix) with 16S rRNA genes as a template. The reverse transcribed products were heat denatured and resolved on TBE-urea polyacrylamide sequencing gels and then scanned on a Typhoon 5 Imager (Cytiva). Secondary structure of S. aureus 16S rRNA was obtained from RNAcentral database (91).
