# Supplementary material for: Hibernation-Promoting Factor Sequesters Staphylococcus aureus Ribosomes to Antagonize RNase R-Mediated Nucleolytic Degradation
Source: mBio. 2021 Jul 13;12(4):e00334-21. doi: 10.1128/mBio.00334-21 (PMC8406268; doi:10.1128/mBio.00334-21)
Supplement: TABLE S1 [file mbio.00334-21-st001.docx]

**Table S1. Strains and plasmids.**

| **Name** | **Genotypes and relevant features** | **Source** |
| --- | --- | --- |
| ***S. aureus***  JE2 | Parental strain, plasmid cured LAC USA300 strain | BEI resources (2) |
| RN4220 | *sau1*^-^, *hsdR*^-^*, mec*^-^, *rsbU*^-^, *agr*^-^, plasmid passage host | ATCC NR-45946 |
| MNY133 | JE2 Δ*hpf*::Km | This study |
| NE471 | JE2 Δ*rnhA*::Erm | BEI resources |
| NE664 | JE2 Δ*mrnC*::Erm | BEI resources |
| NE501 | JE2 Δ*rnr*::Erm | BEI resources |
| NE1936 | JE2 Δ*rnhC*::Erm | BEI resources |
| NE905 | JE2 Δ*rnmV*::Erm | BEI resources |
| MNY186 | JE2 Δ*ybeY*::Erm | This study |
| NR46802 | JE2 Δ*pnpA::*Erm | BEI resources |
| NE1494 | JE2 Δ*rnc*::Erm | BEI resources |
| NE1437 | JE2 Δ*yefM1*::Erm | BEI resources |
| NE1853 | JE2 Δ*yoeB1*::Erm | BEI resources |
| NE1091 | JE2 Δ*yefM2::*Erm | BEI resources |
| NE1117 | JE2 Δ*rae1*::Erm | BEI resources |
| NE1833 | JE2 Δ*mazF*::Erm | BEI resources |
| MNY190 | JE2 Δ*rnmV*::Spc | This study |
| MNY171 | JE2 Δ*rnhA*::Erm, Δ*hpf*::Km | This study |
| MNY173 | JE2 Δ*mrnC*::Erm,Δ*hpf*::Km | This study |
| MNY175 | JE2 Δ*rnr*::Erm, Δ*hpf*::Km | This study |
| MNY176 | JE2 Δ*yhaM*::Erm, Δ*hpf*::Km | This study |
| MNY178 | JE2 Δ*rnhC*::Erm, Δ*hpf*::Km | This study |
| MNY180 | JE2 Δ*rnmV*::Erm, Δ*hpf*::Km | This study |
| MNY181 | JE2 Δ*ybeY*::Erm, Δ*hpf*::Km | This study |
| MNY183 | JE2 Δ*pnpA*::Erm, Δ*hpf*::Km | This study |
| MNY113 | JE2 Δ*rnc*::Erm, Δ*hpf*::Km | This study |
| MNY139 | JE2 Δ*yefM1*::Erm, Δ*hpf*::Km | This study |
| MNY141 | JE2 Δ*yoeB1*::Erm, Δ*hpf*::Km | This study |
| MNY143 | JE2 Δ*yefM2*::Erm, Δ*hpf*::Km | This study |
| MNY145 | JE2 Δ*rae1*::Erm, Δ*hpf*::Km | This study |
| MNY199 | JE2 Δ*mazF*::Erm Δ*hpf*::Km | This study |
| MNY193 | JE2 Δ*rnmV*::Spc, Δ*yhaM::*Erm | This study |
| MNY201 | JE2 Δ*rnmV*::Spc, Δ*rnr::*Erm | This study |
| MNY207 | JE2 Δ*rnmV*::Spc, Δ*rnr::*Erm, Δ*hpf*::Km | This study |
| NE1708 | JE2 Δ*clpP*::Erm | BEI resources |
| NE912 | JE2 Δ*clpY*::Erm (*clpY* a.k.a. *hslU*) | BEI resources |
| ***E. coli***  BL21(DE3) | F^–^ *ompT* *gal* *dcm* *lon* *hsdS_B_*(*r_B_*^–^*m_B_*^–^) λ(DE3 [*lacI* *lacUV5*-*T7p07* *ind1* *sam7* *nin5*]) [*malB*^+^]_K-12_(λ^S^) | Lucigen |
| DC10B | DH10B Δ*dcm* | (3) |
| **Plasmids**  pEPSA5 | *E. coli-S. aureus* shuttle vector, pT5X xylose-inducible promoter, Amp^R^, Cm^R^ | (4) |
| pBT2 | Temperature sensitive*, E. coli-S. aureus* shuttle vector, Amp^R^, Cm^R^ | (5) |
| pBTK | Temperature sensitive, *E. coli-S. aureus* shuttle vector, 1.4-kb *aph-A3* cloned into *SmaI* site of pBT2, Amp^R^, Cm^R^, Km^R^ | (6) |
| pBTE | Temperature sensitive, *E. coli-S. aureus* shuttle vector, 1.2-kb *ermBL-ermB* cloned into *SmaI* site of pBT2, Amp^R^, Cm^R^, Erm^R^ | (6) |
| pMCSG7 | Ligation independent overexpression plasmid, cleavable His tag by TEV, Amp^R^. | (7) |
| pBT2∆*ybeY*::Erm | ~3.2 kb ∆*ybeY*::Erm on pBT2, ts, Amp^R^, Cm^R^ | This work |
| pBT2∆*hpf::*Km | ~3.6 kb ∆*hpf*::Km on pBT2, ts, Amp^R^, Cm^R^ | This work |
| pMCSG7::rnr (WT) | ~ 2.8-kb *rnr* on pMCSG7, Amp^R^, encoding N-terminally 6His-tagged Rnr | This work |
| pMCSG7::rnr (D271N) | ~ 2.8-kb *rnr(*D271N) on pMCSG7, Amp^R,^ encoding N-terminally 6His-tagged Rnr (D271N) | This work |
| pEPSA5::3FLAG_rnr (WT) | ~2.4-kb *flag_rnr* cloned into EcoRI and KpnI sites of pEPSA5, Amp^R^, Cm^R^ | This work |
| pEPSA5::3FLAG_rnr (D271N) | ~2.4-kb *flag_rnr*(D271N) cloned into EcoRI and KpnI sites of pEPSA5, Amp^R^, Cm^R^ | This work |

**SUPPLEMENTAL REFERENCES**

1. Anonymous (2019) RNAcentral: a hub of information for non-coding RNA sequences. *Nucleic Acids Res* 47(D1):D221-D229.

2. Fey PD*, et al.* (2013) A genetic resource for rapid and comprehensive phenotype screening of nonessential *Staphylococcus aureus* genes. *MBio* 4(1):e00537-00512.

3. Monk IR, Shah IM, Xu M, Tan MW, & Foster TJ (2012) Transforming the untransformable: application of direct transformation to manipulate genetically *Staphylococcus aureus* and *Staphylococcus epidermidis*. *mBio* 3(2):e00277-00211.

4. Forsyth RA*, et al.* (2002) A genome-wide strategy for the identification of essential genes in *Staphylococcus aureus*. *Mol Microbiol* 43(6):1387-1400.

5. Bruckner R (1992) A series of shuttle vectors for *Bacillus subtilis* and *Escherichia coli*. *Gene* 122(1):187-192.

6. Fuller JR*, et al.* (2011) Identification of a lactate-quinone oxidoreductase in *Staphylococcus aureus* that is essential for virulence. *Front Cell Infect Microbiol* 1:19.

7. Stols L*, et al.* (2002) A new vector for high-throughput, ligation-independent cloning encoding a tobacco etch virus protease cleavage site. *Protein Expr Purif* 25(1):8-15.
