# Supplementary figures and images for: Hibernation-Promoting Factor Sequesters Staphylococcus aureus Ribosomes to Antagonize RNase R-Mediated Nucleolytic Degradation
Source: mBio. 2021 Jul 13;12(4):e00334-21. doi: 10.1128/mBio.00334-21 (PMC8406268; doi:10.1128/mBio.00334-21)

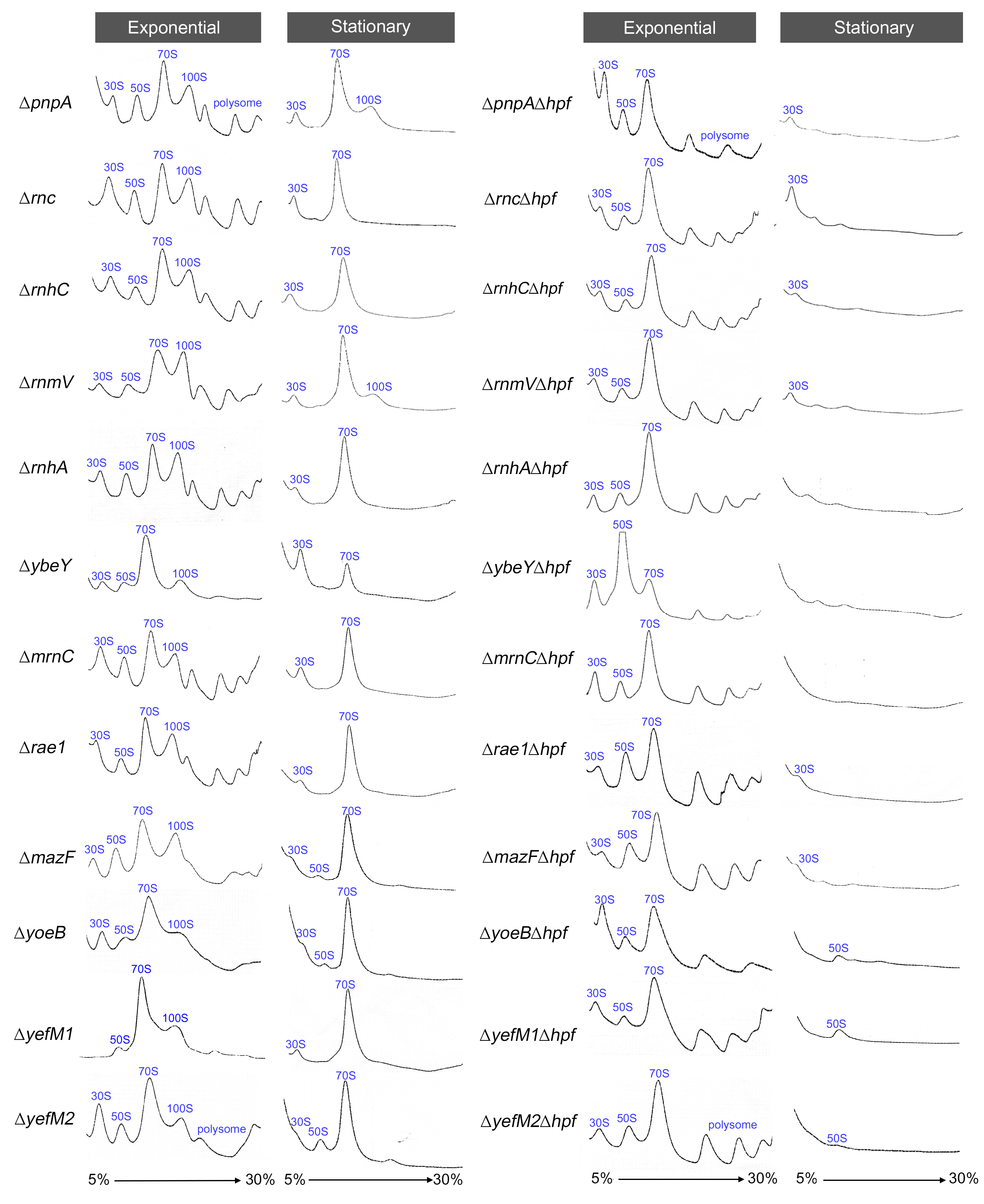

Supplement: FIG S1 [file mbio.00334-21-sf001.tif]

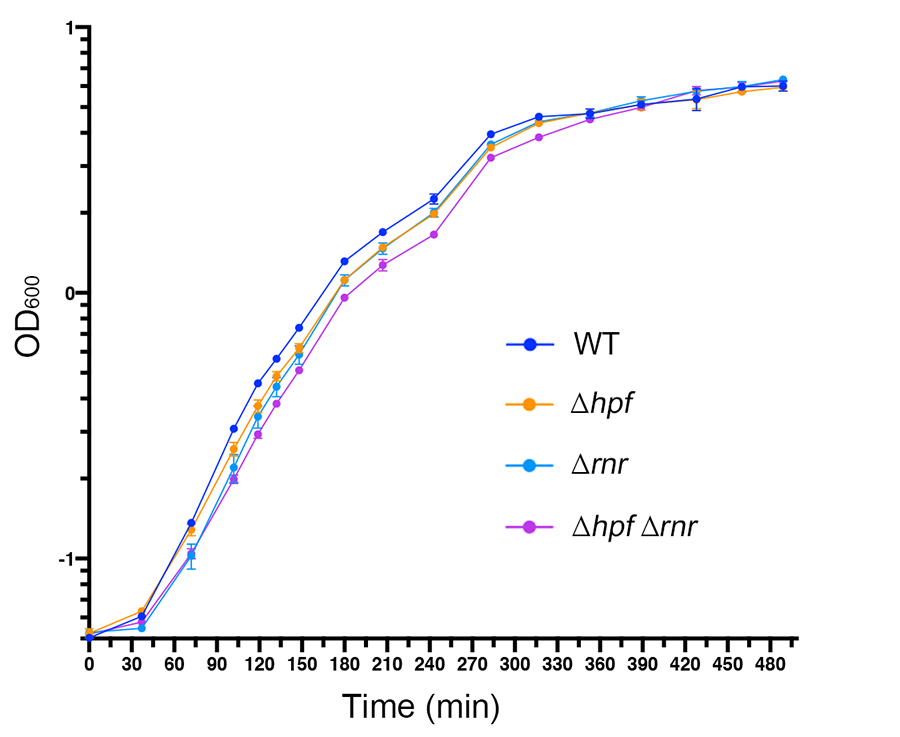

Supplement: FIG S2 [file mbio.00334-21-sf002.tif]

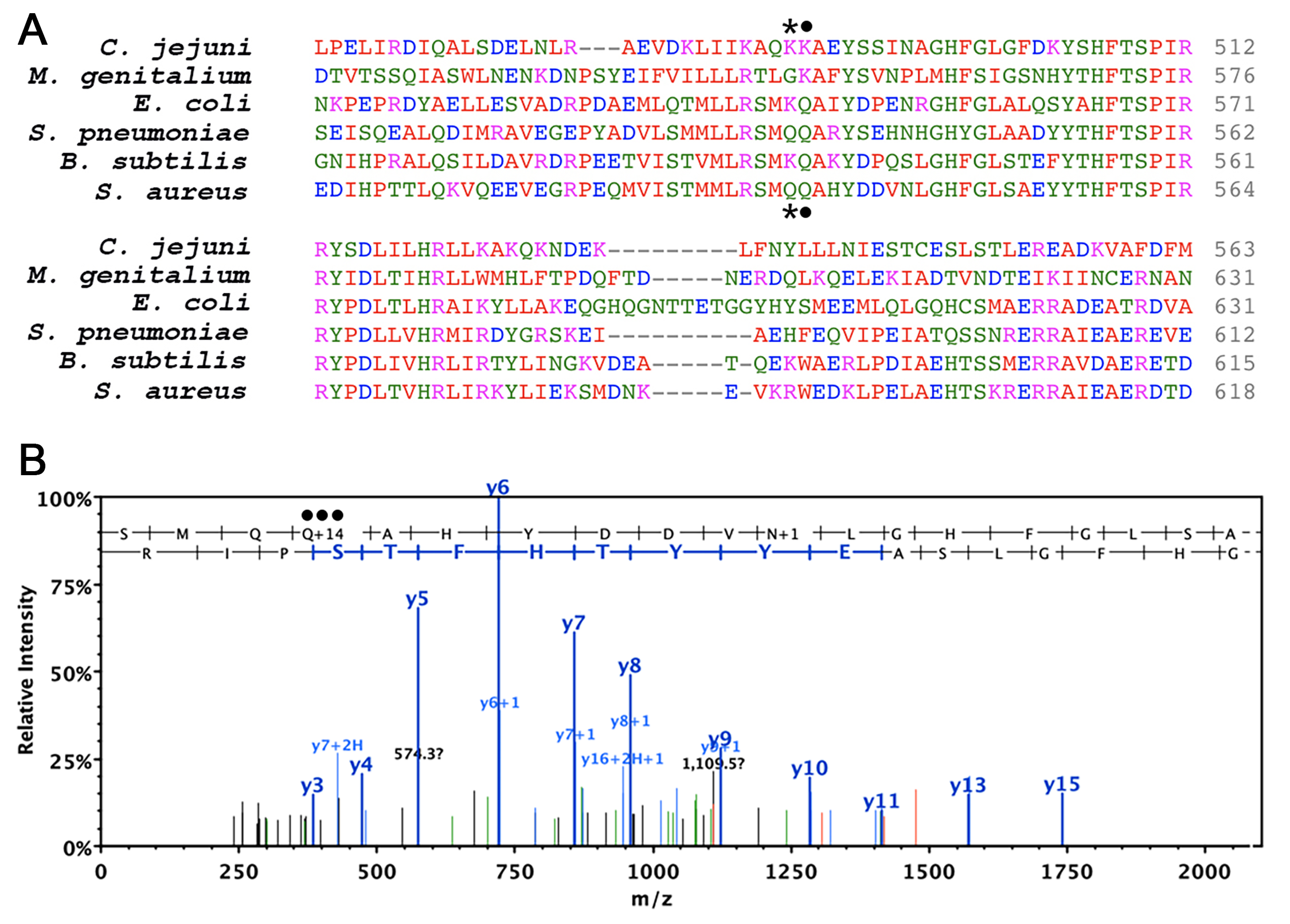

Supplement: FIG S3 [file mbio.00334-21-sf003.tif]

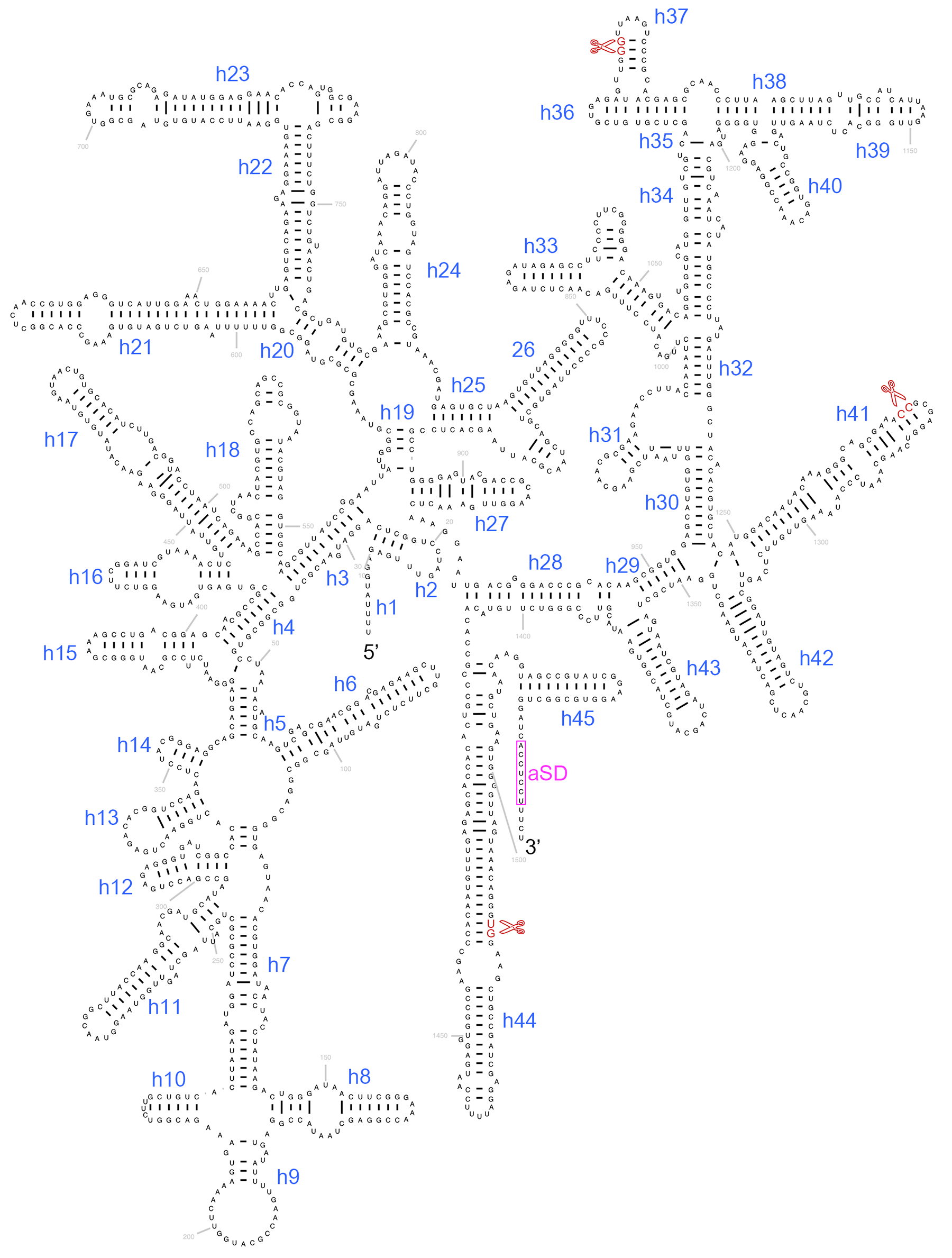

Supplement: FIG S4 [file mbio.00334-21-sf004.tif]
