# Supplementary material for: Hibernation-Promoting Factor Sequesters Staphylococcus aureus Ribosomes to Antagonize RNase R-Mediated Nucleolytic Degradation
Source: mBio. 2021 Jul 13;12(4):e00334-21. doi: 10.1128/mBio.00334-21 (PMC8406268; doi:10.1128/mBio.00334-21)
Supplement: TABLE S2 [file mbio.00334-21-st002.docx]

**Table S2. Primers used in this study.**

| **Primer** | **Sequence (5’-3’)^a^** | **Application** |
| --- | --- | --- |
| P1237 (SacI)  P1238 (SmaI)  P1239  (SmaI)  P1240 (PstI) | AATGAGCTCGTCAATGCAAAGATGTTTAAATCAATCA  TGCCGCGTACTCTGCGCCCGGGATATCTATCGTAAACATTTAATTCTCTCCTTCATA  ATCCCGGGCGCAGAGTACGCGGCATAATTATGAAAAGGTTTAAATATGCACTTGATG  TATACTGCAGTCATCGCCTCGTCCAATTTCCTCATTGGC | Primers to create pBT2∆*ybeY*::Erm for allelic exchange |
| P1241  P1242 | TGATTAGTCTAAAATTGAATCTGCTTT  ACTGGTGTCTTAACATTTTTCAACATTTCTA | To confirm chromosomal ∆*ybeY*::Erm allele |
| P0687  (SalI)  P0688 (SmaI)  P0689  (SmaI)  P0690  (SacI) | ATGTCGACGTCATTTTTACTACGACAATATTAGAACGTG  CCGCGTACTCTGCGTCCCGGGCACATAGTAATCTCTCCTTAAACCTCTTTA TGCCCGGGACGCAGAGTACGCGGTAAATTAAGTTTAAAGCACTTGTGTTTTTGCAC  ATGTGAGCTCGTAAGTAATCAAAACCTAGCTCATTA | Primers to create pBT2∆*hpf*::Km for allelic exchange |
| P0691  P0692 | AATTCATCGTGCAGGGTGTAAATTATA  TATTCTCAATACGCAATTATACTTCCTA | To confirm chromosomal ∆*hpf*::Km allele |
| P1462 (EcoRI)  P1463 (KpnI) | TTAGAATTCGAAAGGAGGGGCATAATG**GATTATAAAGATCATGATGGCGATTATAAAGATCATGATATTGATTATAAAGATGATGATGATAAA**AATTTAAAGCAATCTATAGAAGAGA  TTGGTACCATTCATACTCACCTCATTGCTG | **3×FLAG**-tagged *rnr* |
| P1464  P1465 | GATGCTAAAGACTTAAATGACGCAATTAGTG  CACTAATTGCGTCATTTAAGTCTTTAGCATC | Site-directed mutagenesis of *rnr* (D271N) |
| P1436  P1437 | TACTTCCAATCCAATGCCATGAATTTAAAGCAATCTATAGAA  TTATCCACTTCCAATGTTATATTTTTTCTTACGACGTGCTTTCTTTTTCACA | Ligation-independent cloning of *rnr* into pMCSG7 |
| P1430  P1431 | \| TTCTGTATAATAGATTCAAGTTAGT  TCTGATTTAATCTGTATCAGGCTGA \| \| --- \| | DNA sequencing primers on pEPSA5 |

^a^: restriction enzyme cleavage sites are underlined
