# Supplementary material for: Hibernation-Promoting Factor Sequesters Staphylococcus aureus Ribosomes to Antagonize RNase R-Mediated Nucleolytic Degradation
Source: mBio. 2021 Jul 13;12(4):e00334-21. doi: 10.1128/mBio.00334-21 (PMC8406268; doi:10.1128/mBio.00334-21)
Supplement: TABLE S3 [file mbio.00334-21-st003.docx]

**Table S3. 6-carboxyfluorescein (6-FAM) labeled or unlabeled RNA and DNA oligonucleotides used in this study**

| **Primer** | **Sequence (5’-3’)** | **Application** |
| --- | --- | --- |
| RNA1 | \| ((6-FAM)-GUUGAGAGAGAGAGAGAGUUUG \| \| --- \| \|  \| | In vitro RNase R substrate |
| RNA2 | CUCUCUCAAC | In vitro RNase R substrate, complementary to RNA1 |
| a:16S(1484-1506) | (6-FAM)-ACTTCACCCCAATCATTTGT | Primer extension |
| b:16S(1391-1411) | (6-FAM)-CGGTGTGTACAAGACCCGGGA | Primer extension |
| c:16S(1198-1215) | (6-FAM)-ATGATGATTTGACGTCAT | Primer extension |
| d:16S(927-946) | (6-FAM)-TGTGCGGGTCCCCGTCAAT | Primer extension |
| e:16S(820-839) | (6-FAM)-ACTTAGCACTCATCGTTTAC | Primer extension |
| f:16S(511-531) | (6-FAM)-TGCTGGCACGTAGTTAGCCGT | Primer extension |
| g:16S(1329-1349) | (6-FAM)-TCCAGCTTCATGTAGTCGAGT | Primer extension |
| h:16S(293-315) | (6-FAM)-ATCACCCTCTCAGGTCGGCTATG | Primer extension |
| i:16S(148-170) | (6-FAM)-AGCTCCGGTTTCCCGAAGTTATC | Primer extension |
